# Middle to late Holocene plant cover variation in relation to climate, fire, and human activity in the Songnen grasslands of northeastern China

**DOI:** 10.3389/fpls.2022.1071273

**Published:** 2023-01-09

**Authors:** Honghao Niu, Laurent Marquer, Dorothy Sack, Guizai Gao, Jiangyong Wang, Meng Meng, Dongmei Jie

**Affiliations:** ^1^ School of Geographical Sciences, Northeast Normal University, Changchun, China; ^2^ Department of Botany, University of Innsbruck, Innsbruck, Austria; ^3^ Department of Geography, Ohio University, Athens, OH, United States; ^4^ Key Laboratory of Geographical Processes and Ecological Security in Changbai Mountains, Ministry of Education, Changchun, China; ^5^ Institute for Peat and Mire Research, State Environmental Protection Key Laboratory of Wetland Ecology and Vegetation Restoration, Northeast Normal University, Changchun, China; ^6^ Key Laboratory of Vegetation Ecology, Ministry of Education, Changchun, China

**Keywords:** holocene, pollen, REVEALS (Regional Estimates of Vegetation Abundance from Large Sites) model, 4.2 ka BP event, EASM

## Abstract

**Introduction:**

For future vegetation projections and conservation planning in grassland ecosystems, accurate estimates of past plant cover changes in grassland composition and their responses to the various driving factors are essential. This study quantitatively reconstructs the past regional plant cover in the Songnen grasslands (northeastern China) and explores the relative importance of climate, fire, and human activity on vegetation dynamics.

**Methods:**

For this purpose, the Regional Estimates of Vegetation Abundance from Large Sites (REVEALS) model is applied to three pollen records from two areas, two in the center of the Songnen grasslands and one located in an area marginal to the grasslands.

**Results:**

Results from the most reliable REVEALS scenarios show that from the mid-Holocene, steppe (mean cover 40.6%) and dry steppe (mean cover 54.2%) alternately dominated the central part of the Songnen grasslands while the marginal grasslands were mainly characterized by alternating broadleaved forests (mean cover 26.3%), coniferous forests (mean cover 41.9%) and dry steppes (mean cover 30.1%).

**Discussion:**

By comparing the plant cover results with previous published regional climate, fire and human activity records, the results show that long term vegetation dynamics were mainly driven by East Asia Summer Monsoon (EASM) and the related precipitation variations, but was also affected by fire frequency and human activity. Moreover, vegetation evolution was sensitive to abrupt cooling events including the 4.2 ka BP and stacked ice-rafted debris (IRD) events; the change from steppe to dry steppe, for example, was driven by these abrupt climate changes. Fire events can alter the original vegetation stability allowing the vegetation to respond rapidly to climate changes while human activity merely has limited influence on vegetation changes.

## 1 Introduction

Grasslands represent about 40% of the Chinese land cover. These landscapes are expected to change significantly over the next few decades because of ongoing global warming and an increase in human activities such as overgrazing and land reclamation (Wang et al., 2007; IPCC, 2014). These climate and land-use changes will undoubtedly affect grassland ecosystems *via* alterations in biogeochemical cycles, species composition, spatial distribution of plant communities, and other characteristics (Zhou et al., 2005; Zuo et al., 2009; Zhai et al., 2021). In addition, human impacts on grasslands might accelerate the degradation of these ecosystems and further advance desertification. Such alterations would hamper economic development in the area in the future (Niu et al., 2008; Ren et al., 2017).

In the northeastern part of China, the Songnen grasslands, also known as Songnen sandy lands, occupy a critical location at the transition between the eastern margin of the inner Mongolia temperate grasslands, the Horqin sandy land, and the forests of the Greater Khingan Mountains (Daxinganling) (Song et al., 2012). During the past few decades, intense human influences have resulted in deterioration and severe salinization of the Songnen grasslands (Wang et al., 2009; Sun et al., 2013). From 1980 to 2015, the extent of saline lands in the region increased approximately 20% and most grasslands are expected to be transformed to saline lands in the near future (Wang & Li, 2018). To reverse this increasing land salinization, a series of ecological protection policies would have to be established and implemented (Doren et al., 2009; Cao, 2011). In addition, the understanding of the long-term perspective of change in vegetation composition and dynamics and their driving forces is necessary to provide insights into the potential effects of future grassland management strategies for natural vegetation restoration (Li et al., 2018).

Fossil pollen records are reliable archives to provide past vegetation information from several centuries to millennia and from local to regional spatial scales (e.g. Davis and Brubaker., 1973; O’Dwyer et al., 2021; Chen et al., 2023). In the Songnen grasslands, pollen analyses have been used to reconstruct late Pleistocene changes in the regional vegetation (Li, 1991). Those studies were conducted on sand dune palaeosols. Results suggested that the grasslands were dominated by *Artemisia* and Chenopodiaceae plants during the Holocene (Li, 1991; Qiu et al., 1992; Li & Lv, 1996). However, those findings were challenged by the vegetation pattern proposed in recent research based on phytoliths, which shows Poaceae-dominant grasslands as a major vegetation component in the region since the early Holocene (Li et al., 2018). Several possible explanations could account for this discrepancy. First, phytoliths are essentially deposited *in situ* and therefore a phytolith assemblage reflects the local vegetation rather than regional plant communities (Strömberg et al., 2018), whereas a pollen assemblage is assumed to represent a mix of local and regional plant taxa. Second, pollen studies from palaeosols have low temporal resolution, and the pollen materials are poorly preserved in sandy sediments (Alexandre et al., 1997; Boyd, 2005). Third, the phytolith and pollen data are typically discussed for each site individually, which may be insufficient for representing the general regional vegetation patterns (Nolan et al., 2018). Fourth, pollen data are discussed in terms of pollen proportion and no modelling schemes have been applied to correct the inter-taxonomic differences in production, dispersal, and deposition of pollen. This means that plants characterized by high pollen productivities and high transportation abilities are overestimated in the pollen proportion compared to the “real” vegetation (e.g., Sugita, 1994; Xu et al., 2007; Gaillard et al., 2008; Marquer et al., 2014; Marquer et al., 2020). To reduce the uncertainties associated with this fourth issue, the temporal resolution of the pollen archives must be improved and pollen-based vegetation modelling approaches should be performed to consider the production, dispersal, and deposition of the different pollen types in the vegetation reconstructions.

One of the most used pollen-based vegetation modelling approach to date is the REVEALS (Regional Estimates of Vegetation Abundance from Large Sites) model (Sugita, 2007; Hellman et al., 2008b). This model corrects for the pollen taphonomical issues mentioned in the previous paragraph by using input parameters that include relative pollen productivity estimates (RPPs), fall speeds of pollen (FSPs), pollen counts for specific time windows, size of the sedimentary basin, wind speed, dispersal models, and other factors. The REVEALS model quantitatively reconstructs plant cover for a given spatial extent (commonly 50–100 km); the exact extent of a reconstruction is chosen when running the model. Initially, one of the major assumptions for the use of REVEALS is that the sedimentary basin should be a large lake, i.e., a water surface area larger than 50 ha. However, REVEALS can also provide reliable estimates of plant cover for a group of small sites with various basin types including small lakes, bogs, and marshes (Trondman et al., 2016). Thus far, REVEALS has been widely applied to reconstruct Holocene land cover in different regions, especially Europe and China (e.g., Marquer et al., 2014; Trondman et al., 2015; Marquer et al., 2017; Cao et al., 2019; Li et al., 2020; Githumbi et al., 2022). REVEALS has rarely been applied to semiarid regions such as the Songnen grasslands.

Since the mid-Holocene, the global climate has experienced a series of abrupt-change events, such as the 4.2 ka BP and IRD events (Bond et al., 2001; Weiss & Bradley, 2001). These abrupt events have been mostly reported from the northern hemisphere including North America, Europe, and Asia (Booth et al., 2005; Drysdale et al., 2006; Berkelhammer et al., 2012). Previous research has shown that in northern China these events were characterized by a sudden shift from warm and wet to cold and dry climate conditions and have played significant roles in regional land-cover changes and collapse of civilizations (Ran & Chen, 2019; Scuderi et al., 2019). The response of the grassland ecosystem to these events, however, has not been fully studied.

The present study aims to reconstruct changes in the regional vegetation cover in the Songnen grasslands during the middle to late Holocene and to explore their driving forces. Three pollen records were used for this purpose. After evaluating the validity of the REVEALS model for Songnen grasslands, we selected the most reliable scenarios to reconstruct the mid to late Holocene vegetation changes in the Songnen grasslands. Finally, the outcomes from the vegetation reconstructions are discussed in the context of the past regional climate, fire records, and human activity to explore their main driving mechanisms temporal and spatially.

## 2 Study region

The study region consists of the Songnen grasslands (44°45′–48°20′N, 120°40′–126°00′E), which are located at the eastern margin of the inner Mongolia temperate steppe in Northeast China. Controlled by the East Asian monsoon system, the region is characterized by a semiarid climate with an average annual temperature of 3.5°C to 5.0°C from north to south, and an average annual precipitation of 360 mm to 480 mm from west to east. At least 70% of the total annual precipitation falls during the short summers resulting from the East Asian Summer Monsoon (EASM), which brings warm and humid air from the Pacific Ocean to the study region. The region is characterized by long winters generally starting in October/November and ending in May/June; dominant northwesterly winds in winter bring cold and dry air from central Asia (Li, 1994). Evaporation rates are high, about 1600 mm/year, and wind speeds vary from 3–6 m/s (Xiao, 1995; Jin et al., 2012).

The main geomorphological features of the region consist of sand dunes and interdune lowlands. Shrubs and xerophytic plants such as *Artemisia*, Chenopodiaceae, and Compositae grow on the sand dunes, while Poaceae (e.g., *Leymus chinensis, Stipa baicalensis*, and *Arundinella hirta*) dominate the interdune lowlands. Until recently, many saline lakes were distributed sporadically in the central part of the Songnen grasslands. In recent years, shrinkage of these lakes owing to global warming and intensified human activities has created saline lands that are characterized by xerophytic plant compositions similar to those found on the sand dunes.

**Figure 1:**
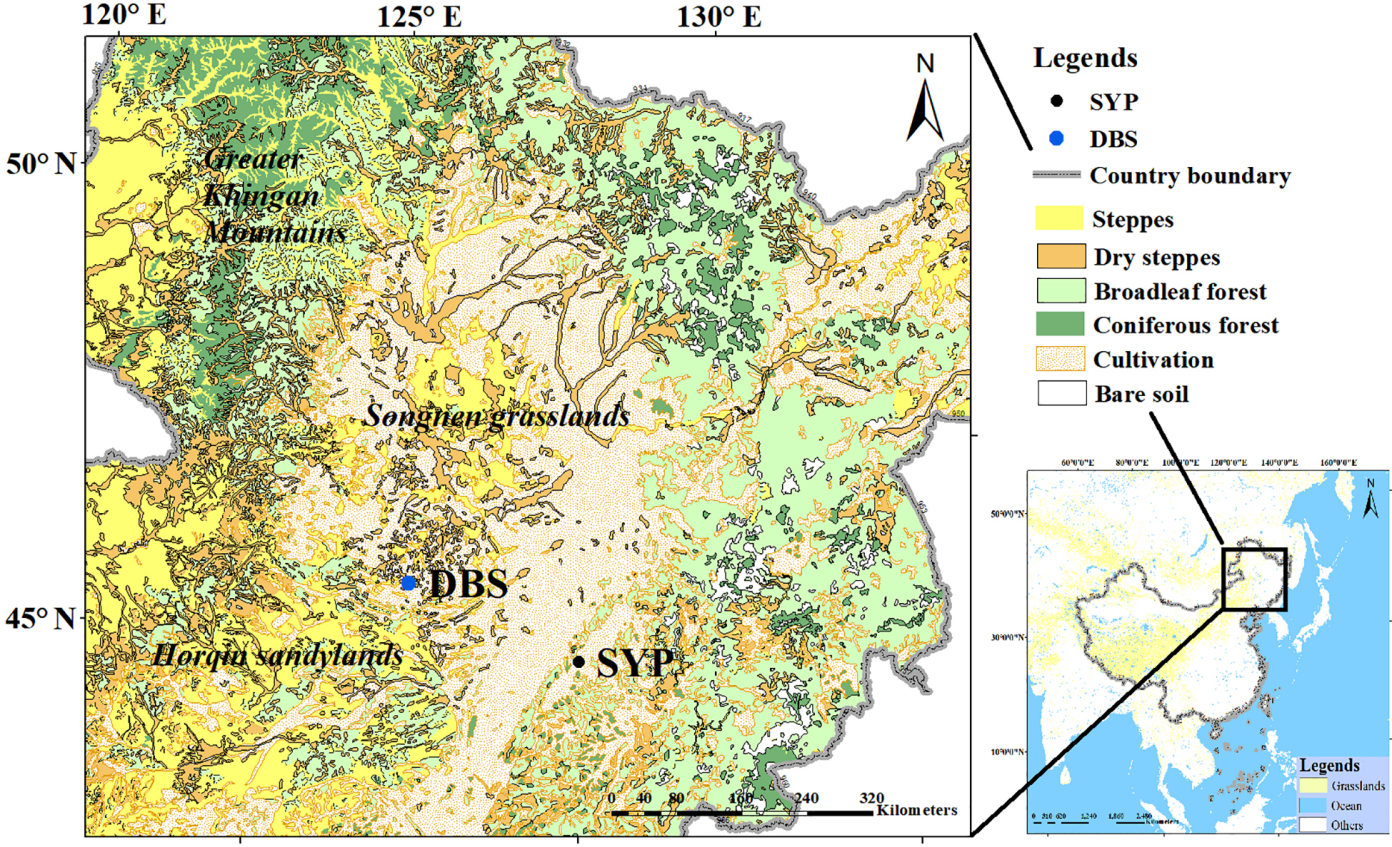
Present vegetation of the study area. The location of the pollen records used in this study are shown: Dabusu lake and peat sediments (DBS) and Shuangyang peatland sediments (SYP). Grassland cover at a continental scale appears in yellow on the map of China (right panel).

Lake Dabusu lies in the south-central part of the Songnen grasslands. The lake has a surface area of approximately 37 km^2^ with a water depth varying seasonally between 0.5 and 1.5 m (**Figure 1**). Sedges and other helophytes grow on the peatlands formed on the lake’s northern shore. Fields of rice and corn have been planted near the lake over the last decades.

In the southeastern margin of Songnen grasslands, the Shuangyang peatlands formed on a partially enclosed depression in the valley on the north side of the Xiaoyingzi River (**Figure 1**). Because of a more humid climate in this area, coniferous and broadleaved mixed forests, including *Pinus*, *Betula*, and *Quercus*, are present in the eastern part, while forest-steppe and cultivated farmland are the main vegetation types in the west.

## 3 Materials and methods

### 3.1 Pollen data

This study is based on three temporally continuous pollen records located in the Songnen grasslands-one from lake and two from peat sediments (**Table 1**; **Figure 1**). The pollen records from Lake Dabusu (DBSL) are published in Jie (2000) and Jie et al. (2001). Records from peat sediments include the recently published Dabusu peat (DBSP) data (Niu et al., 2022) and those from the Shuangyang peat (SYP) published by (Chen, 1997). Dating information is provided in **Table 2**, and pollen counts from all sites are provided in **Supplementary Material S1**.

**Table 1:** Metadata (we choose the modern basin size for REVEALS modelling in this study).

Site names	Lat. (°)	Long.(°)	Archive type	Basin radius (m)	Pollen count	Number of dating controls	Chronology;end date (yr)	References
DBSL	123.22	44.83	Lake	5000	Raw	4	8761	Jie (2000) and Jie et al. (2001)
DBSP	123.21	44.84	Peat	1500	Raw	6	7340	Niu et al., 2022
SYP	125.56	43.61	Peat	800	Raw	4	6485	Chen, 1997

**Table 2:** Dating information.

Site names	Core no.	Depth (cm)		Lab. code	Materials	AMS 14C yr BP	Uncertainty	2σ-range calibrations (cal. yr BP) with probability	Median age, cal. yr BP
**DBSP**	DBSP-1	35	NENUR 10479		Plant residues	215	35	135-225 (50.3%)	190
	DBSP-2	73	NENUR 10480		Plant residues	1300	40	1170-1300 (90.9%)	1225
	DBSP-3	131	NENUR 10481		Bulk organic matter	2625	40	2705-2800 (87.8%)	2750
	DBSP-4	195	NENUR 10483		Bulk organic matter	5030	50	5655-5905 (93.4%)	5785
	DBSP-5	225	NENUR 10484		Plant residues	4675	50	5310-5485 (85.7%)	5405
	DBSP-6	258	NENUR 10485		Bulk organic matter	6320	60	7155-7365 (85.1%)	7240
	DBSP-7	290	NENUR 10486		Bulk organic matter	6260	60	7145-7351 (60.3%)	7180
**DBSL**	DBSL-1	120	SNQD14C1		Bulk organic matter	1470	85	1179-1537 (93.3%)	1265
	DBSL-2	460	SNQD14C2		Bulk organic matter	4230	100	4441-5209 (93.3%)	4511
	DBSL-3	800	SNQD14C3		Bulk organic matter	7225	140	7782-8355 (95.4%)	7782
	DBSL-4	960	SNQD14C4		Bulk organic matter	9735	125	10695-11605 (92.7%)	11107
**SYP**	SYP-1	73	NENU5		Bulk organic matter	1630	65	1375-1696 (89.7%)	1503
	SYP-2	189	NENU6		Bulk organic matter	3100	75	3076-3459 (92.5%)	3298
	SYP-3	289	NENU7		Bulk organic matter	4580	80	4974-5550 (92.2%)	5253
	SYP-4	381	NENU8		Bulk organic matter	6745	90	7430-77501 (92.5%)	7605

### 3.2 REVEALS reconstructions

#### 3.2.1 RPPs and FSPs

RPPs and FSPs are critical input parameters for REVEALS model and they vary among regions and continents because of differences in plant species, climate, and land use (e.g. Andersen, 1970; Sugita, 2007; Gaillard et al., 2008; Mazier et al., 2012; Bunting et al., 2013; Qin et al., 2020). In the present study, to find suitable RPP and FSP data for the vegetation reconstructions of the Songnen grasslands, three previous published RPPs and FSPs datasets were used for model testing (e.g., Mazier et al., 2012; Li et al., 2018; Wieczorek and Herzschuh, 2020; Zhang et al., 2021b; **Table 3**). Li et al. (2018) first reviewed the RPPs and FSPs obtained for China to propose a set of standardized estimates (i.e., estimates related to Poaceae) for the application of REVEALS for the country as a whole. (Zhang et al., 2021a) provided another set of RPPs and FSPs based on data obtained over Eurasia, mainly from Europe and China. Each of these different sets of RPPs and FSPs have their own advantages and disadvantages (Zhang et al., 2021a). Wieczorek and Herzschuh (2020) recently made available RPPs and FSPs for China derived from their major synthesis. In the present study, the three sets of RPPs and FSPs suggested by Li et al. (2018), Zhang et al. (2021a), and Wieczorek and Herzschuh (2020) were used, with each dataset representing one REVEALS scenario (total of three scenarios: S1, S2, and S3; **Table 3**). In addition, the average values of RPPs and FSPs from these three data sets were calculated to create a fourth scenario (S4).

**Table 3:** Relative pollen productivity estimates (RPPs) with their standard errors (SEs) and fall speeds of pollen (FSPs) for the 18 pollen types that were used in this study.

Pollen types	RPPs and SEs					FSPs(m s-1)				
	S1	S2	S3	S4	Europe	S1	S2	S3	S4	Europe
Herbs/Shrubs										
*Artemisia*	21.15 ± 0.56	15.11 ± 0.37	14.79 ± 0.30	17.01 ± 0.40	3.48 ± 0.20	0.01	0.01	0.01	0.01	0.03
Compositae	4.4 ± 0.29		3.8 ± 0.15280707	4.1 ± 0.22		0.03		0.03	0.03	
Chenopodiaceae	4.46 ± 0.68	4.95 ± 0.57	7.57 ± 0.64	5.66 ± 0.62	4.28 ± 0.27	0.01	0.02	0.01	0.01	0.02
Poaceae	1.00 ± 0.00	1.00 ± 0.00	.1.00 ± 0.00	1.00 ± 0.00	1 ± 0.0	0.02	0.03	0.02	0.02	0.04
*Ephedra*	1.25 ± 0.18	1.11 ± 0.16		1.18 ± 0.17				0.02	0.02	
Ranunculaceae	7.77 ± 1.56	5.34 ± 0.96	7.86 ± 2.65	6.99 ± 1.72	1.96 ± 0.36	0.01	0.01	0.01	0.01	0.01
Rosaceae	0.22 ± 0.09	0.53 ± 0.07	0.53 ± 0.049	0.42 ± 0.06		0.01	0.02	0.02	0.01	
Trees										
*Pinus*	18.37 ± 0.48	13.75 ± 0.58	17.49 ± 0.46	16.53 ± 0.5	6.38 ± 0.45	0.04	0.04	0.03	0.03	0.03
*Abies*		6.88 ± 1.44		6.88 ± 1.44	6.88 ± 1.44		0.01		0.01	0.12
*Picea*		2.55 ± 0.04	29.4 ± 0.87	15.97 ± 0.45	2.62 ± 0.12		0.06	0.08	0.07	0.06
*Betula*	12.42 ± 0.12	10.26 ± 0.12	11.28 ± 0.16	11.32 ± 0.13	9.07 ± 0.10	0.01	0.02	0.02	0.02	0.02
*Carpinus*		1.64 ± 0.07		1.64 ± 0.07	3.55 ± 0.43	0.03			0.03	0.04
*Alnus*		9.86 ± 0.09		9.86 ± 0.09	9.07 ± 0.10		0.02		0.02	0.02
*Quercus*	5.19 ± 0.07	4.27 ± 0.04	2.5 ± 0.054	3.98 ± 0.05	5.83 ± 0.15	0.02	0.03	0.02	0.02	0.04
*Salix*		1.21 ± 0.04		1.21 ± 0.04	1.22 ± 0.11		0.03		0.03	0.02
*Tilia*	0.65 ± 0.11	0.85 ± 0.09	0.4 ± 0.1	0.63 ± 0.1	0.80 ± 0.03	0.03	0.03	0.03	0.03	0.03
*Ulmus*	4.13 ± 0.92	5.29 ± 0.80	2.24 ± 0.46	3.88 ± 0.72	1.27 ± 0.05	0.02	0.03	0.02	0.02	0.03
Juglans	7.69 ± 0.24	4.83 ± 0.18	3.28 ± 0.11	5.26 ± 0.17	2.35 ± 0.11	0.03	0.03	0.03	0.03	0.06

Several sets of published RPPs and FSPs are shown, i.e., those of Li et al. (2018; S1), Zhang et al. (2021; S2), and Wieczorek and Herzschuh (2020; S3). A fourth set (S4) of RPPs and FSPs was obtained by averaging those for the S1, S2, and S3 data sets. RPPs and FSPs that have been published for Europe (Mazier et al., 2012) are also shown for comparison. All RPPs are related to Poaceae.

The most dominant 18 pollen types in the DBSL, DBSP and SYP records are selected in the present study for our REVEALS reconstructions. For DBSP, the pollen types included in S1, S2, S3, and S4 (**Table 3**) represent 73%, 90%, 68%, and 95%, respectively, of the original terrestrial pollen types from the DBSP core (e.g. see **Supplementary Material S1**); for SYP the values are 70%, 88%, 70%, and 94% and for DBSL they are 85%, 71%, 71%, and 85%. Note that the RPP value of *Sanguisorba* (24.07; Li et al., 2018) is too high and certainly questionable, and therefore was omitted from the REVEALS runs.

#### 3.2.2 Other REVEALS inputs and set ups

Except for RPPs and FSPs, pollen counts, basin size, wind speed, dispersal model, and Z max distances are all necessary parameters for REVEALS runs. Uncertainty in the REVEALS estimates decrease with increasing pollen counts. Here, the REVEALS model was run for a minimum counts of 500 pollen grains per time window. Considering this threshold, the data allowed all pollen counts to group within time windows of 300 years (25 time windows) over the last 7500 cal. yr BP. Basin size is also a key input for the model runs. In this study, basin size for the three pollen profiles used for REVEALS are listed in **Table 1**. With a large site designated as one having an area greater than 50 ha (e.g., Trondman et al., 2015), one large lake and two large bogs were used in this study. The notion that large bogs are not considered for REVEALS applications (e.g., Marquer et al., 2014; Trondman et al., 2016) is addressed in the discussion part of the results section of this paper. In addition, a specific wind speed should be chosen for the REVEALS application. Previous studies in China and Europe commonly used a constant wind speed of 3 m/s because this value is considered reasonable in neutral atmospheric conditions for large regions (e.g., Marquer et al., 2014; Trondman et al., 2015; Marquer et al., 2017; Cao et al., 2019; Li et al., 2020). According to the wind regime in the Songnen grasslands, 3 m/s and 6 m/s wind speeds would be relevant, thus REVEALS was run for these two alternatives. Note that a Gaussian Plume Model (GPM) was used as a dispersal model scheme as no RPP values based on other dispersal modelling schemes are available so far. Pros and cons of using a GPM model for the REVEALS runs can be found in Marquer et al. (2020). Finally, four Z max distances were selected for these scenarios: 10, 20, 50, and 100 km. This means that the vegetation was reconstructed within a radius of 10 to 100 km around the pollen record sites.

### 3.3 Evaluation of REVEALS performances

Before evaluation of REVEALS performance, we classified the pollen records used in this research into two subregions: a central region (DBSL and DBSP) and a marginal region (SYP).

#### 3.3.1 Classification of pollen types into vegetation categories

**Figure 2:**
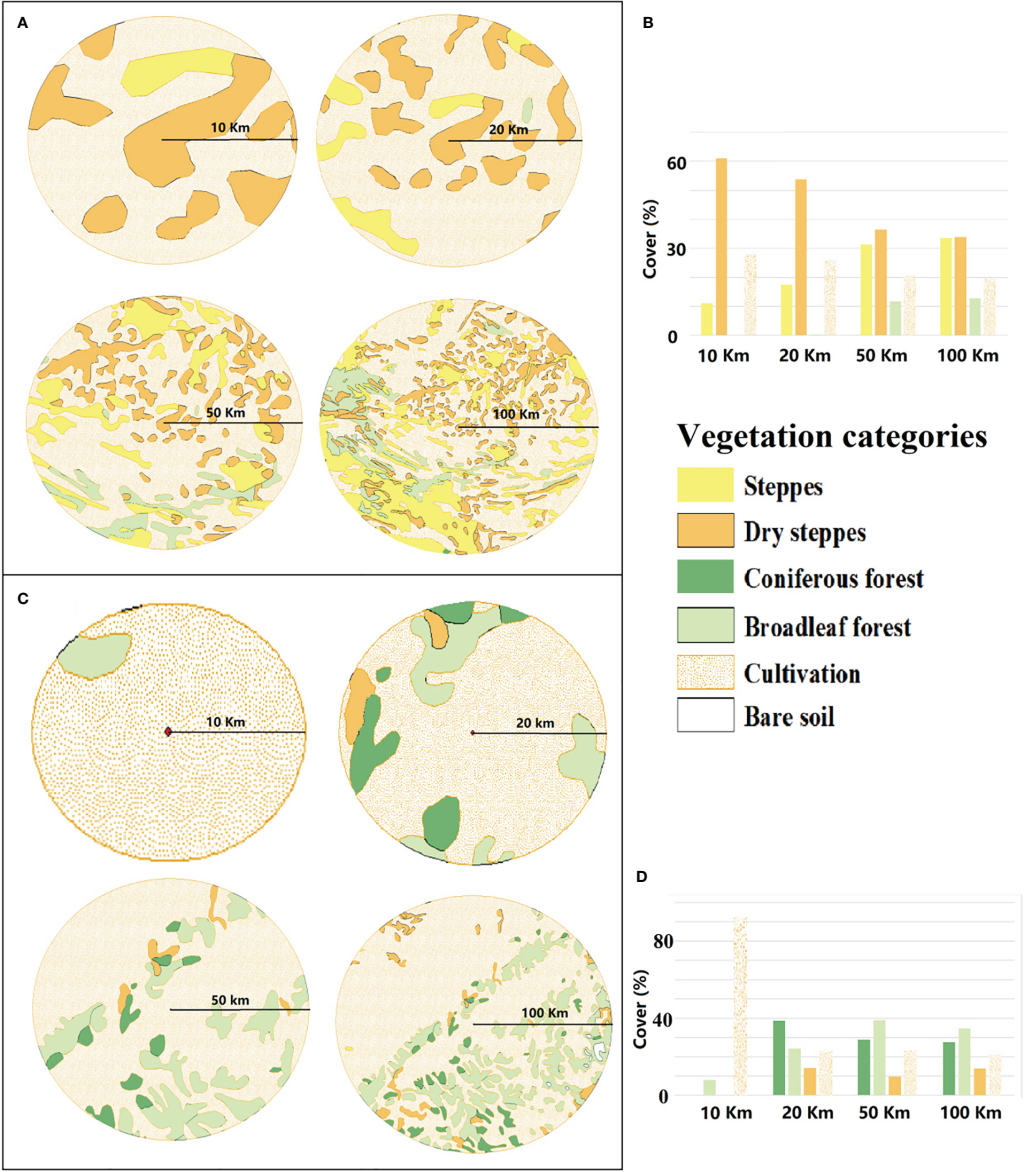
Vegetation maps (http://www.geodata.cn) for extended radii of 10, 20, 50, and 100 km from the pollen record site for central and marginal regions. **(A)**. Vegetation maps for central region. **(B)**. Percentage cover of the different vegetation categories of central region. **(C)**. Vegetation maps for marginal region. **(D)**. Percentage cover of the different vegetation categories of marginal region.

The Songnen grasslands are dominated by the four natural vegetation categories: steppe, dry steppe, broadleaved forest, and coniferous forest (**Figures 1**, **2**). A large part of the study region is cultivated and agriculture represents the major land cover within about 10 km of the pollen record site of marginal region. The main natural vegetation types within a radius of 20 km from the pollen record sites of central region are steppe and dry steppe. From a radius of 50 km to at least 100 km from the central region pollen site, broadleaved forest cover increases. Within marginal region, only broadleaved forests are observed within a radius of 10 km but both coniferous and broadleaved forests dominate the natural regional vegetation from a radius of 20 to at least 100 km; dry steppes are distributed sporadically across marginal region.

To compare REVEALS reconstructions with existing vegetation maps, the pollen types were classified into the four vegetation categories, see above (**Table 4**). Cultivated vegetation was not considered in the comparison because REVEALS would estimate land use based on the *Cerealia* pollen type which would not represent cultivated rice and corn. This limitation is discussed later in the paper.

**Table 4:** Classification of the pollen types into vegetation categories.

	Vegetation categories				
	Steppe	Dry steppe	Coniferous forest	Broadleaved forest	
**Pollen types**	Poaceae	*Artemisia*	*Pinus*	*Betula*	*Salix*
		Compositae	*Abies*	*Carpinus*	*Tilia*
	Ranunculaceae	Chenopodiaceae	*Picea*	*Alnus*	*Ulmus*
	Rosaceae	*Ephedra*		*Quercus*	Juglans

#### 3.3.2 Comparisons of REVEALS estimates with regional vegetation maps

Outcomes from all REVEALS scenarios (e.g., S1 with 3 m/s wind speed) for the most recent time window are compared to the regional vegetation maps (**Figure 2**) derived from the vegetation map of China for the year 2018 (i.e. http://www.geodata.cn). The REVEALS reconstructions within a Z max of 10, 20, 50, and 100 km for the first time window are assessed with respect to regional vegetation maps of the same radii. Although this comparison has limitations because of comparing one year (the year 2018) with the past 300 years, the natural vegetation has not changed dramatically during the last 300 years in this region (Li et al., 1982; Zhang, 2017; Yu, 2017). The comparison can, therefore, still provide insights for evaluating the spatial extent of REVEALS reconstructions. Arcgis 10.5 was used to create the maps and for all related calculations and the detailed procedure can be seen in **Supplementary Material S2**-**Text 1**.

### 3.4 Data processing

To explore the driving forces of the Songnen grasslands vegetation dynamics, several published regional and continental climate datasets, paleofire records, and human activity index were studied. The climate datasets include an EASM index based on results of multiproxy analyses from 11 lakes in central Asia (Chen et al., 2008), the stalagmite δ^18^O records from Nuanhe and Lianhua caves, both in the region adjacent to the Songnen grasslands on the south (Cosford et al., 2009; Wu et al., 2011), and a Holocene stacked IRD events in North Atlantic (Bond et al., 2001). Paleofire datasets include a black carbon concentration records from Tianchi lake (Pang et al., 2021) adjacent to the DBSL and a fire frequency records based on charcoal records from GST peatlands (Meng et al., 2020) close to the SYP. Human activity records were based on the density of archaeological radiocarbon ages in northeast China (Wang et al., 2021).

To assess the relative importance of climate, fire, and human activities on vegetation dynamics during the mid-Holocene, Variation partitioning was performed by using the *varpart ()* function in the “vegan” package of R (Oksanen et al., 2020).

## 4 Results

### 4.1 REVEALS estimates versus regional vegetation maps

We tested the REVEALS performance for both central and marginal regions by using different Z max (10, 20, 50, and 100 km) and wind speeds (3m/s and 6 m/s) under four scenarios (S1, S2, S3 and S4, respectively). After comparing each REVEALS results with corresponding modern vegetation maps, we found that for the central region, with 6 m/s wind speeds, REVEALS estimates were closer to the results calculated from maps with the radius of 50 km for S2, S3, and S4, in particular. For marginal region, when a wind speed of 3 m/s was used, REVEALS estimates were closer to the results calculated from the 20 km map for S4, in particular; however, all vegetation categories are slightly overestimated. All the detailed results of REVEALS estimates versus regional vegetation maps have been shown in **Supplementary Material S2**-**Text 2**.

### 4.2 REVEALS-based vegetation reconstructions

Based on the above results and the **Supplementary Material S2**-**Text 2**, we generated REVEALS estimates for all other time windows using S4, which considers the mean of all RPPs and FSPs and which appears to be a good compromise among all potential alternatives. Regarding wind speed alternatives, 6 m/s was selected for modeling central region and 3 m/s for marginal region.

#### 4.2.1 Central region

**Figure 3:**
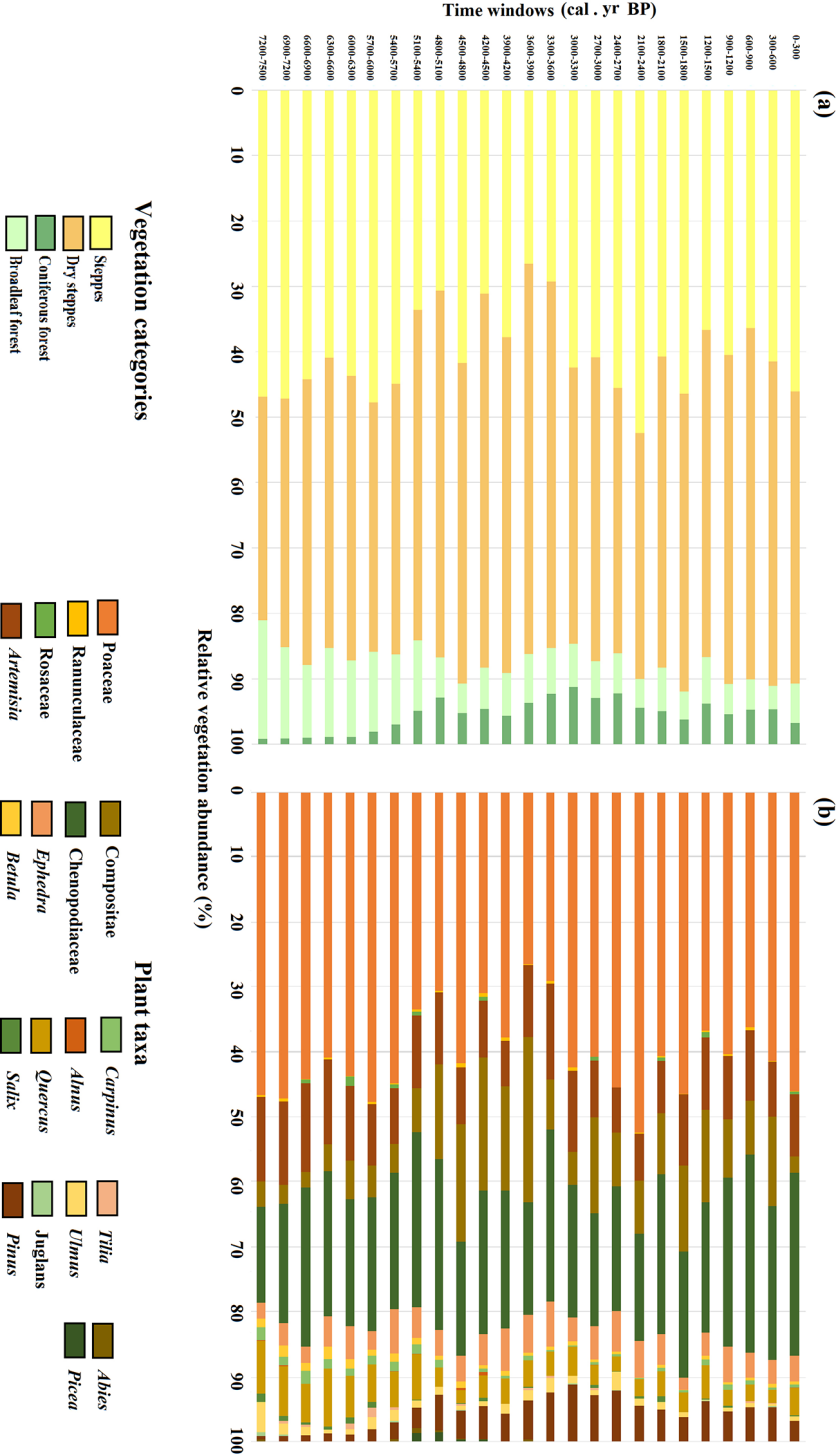
Past vegetation reconstructions based on REVEALS using S4 with a wind speed of 6 m/s for the central region. The four vegetation categories **(A)** and individual plant taxa **(B)** are shown.

Central region has been characterized by steppe and dry steppe since the mid-Holocene (**Figure 3**). The major components of the steppe are Poaceae (mean cover 40.6%). The mean cover of steppe was approximately 45% from 7200 to 5400 cal. yr BP, and then decreased to about 32% between 5400 and 3300 cal. yr BP. The steppe cover increased after 3300 until 1800 cal. yr BP then decreased between 1800 and 900 cal. yr BP. After 900 cal. yr BP and continuing to the present, steppe vegetation increased again to what are relatively high values within the entire sequence with an average cover of 41%.

The plant taxa that dominated the dry steppe were Chenopodiaceae (mean cover 21.7%), *Artemisia* (mean cover 10.3%), Compositae (mean cover 9.7%), and *Ephedra* (mean cover 4.4%). These were the dominant vegetation types of central region for most of the last 7500 years. The extent of dry steppe was lowest during the period 7200–5400 cal. yr BP with a mean cover of 40%. Dry steppe increased to its highest value from 5400 to 3300 cal. yr BP with a mean cover of 52.6%. Coverage of dry steppe decreased from 3300 to 2100 cal. yr BP with a mean cover of 41.7%, then rose again until the present with a mean cover of 48.7%.

Compared to steppe and dry steppe, the extent of broadleaved and coniferous forests has been relatively low, i.e., below 20%, for the last 7500 years. Broadleaved forest achieved its greatest cover in the mid-Holocene until ca. 5100 cal. yr BP, and then decreased to relatively low levels, where it remains today. The cover of coniferous forest, on the other hand, was very low during the mid-Holocene then increased after ca. 5400 cal. yr BP; its maximum cover occurred between 3900 and 2700 cal. yr BP. *Quercus* was the main component of the broadleaved forest when *Pinus* dominated the coniferous forest.

#### 4.2.2 Marginal region

**Figure 4:**
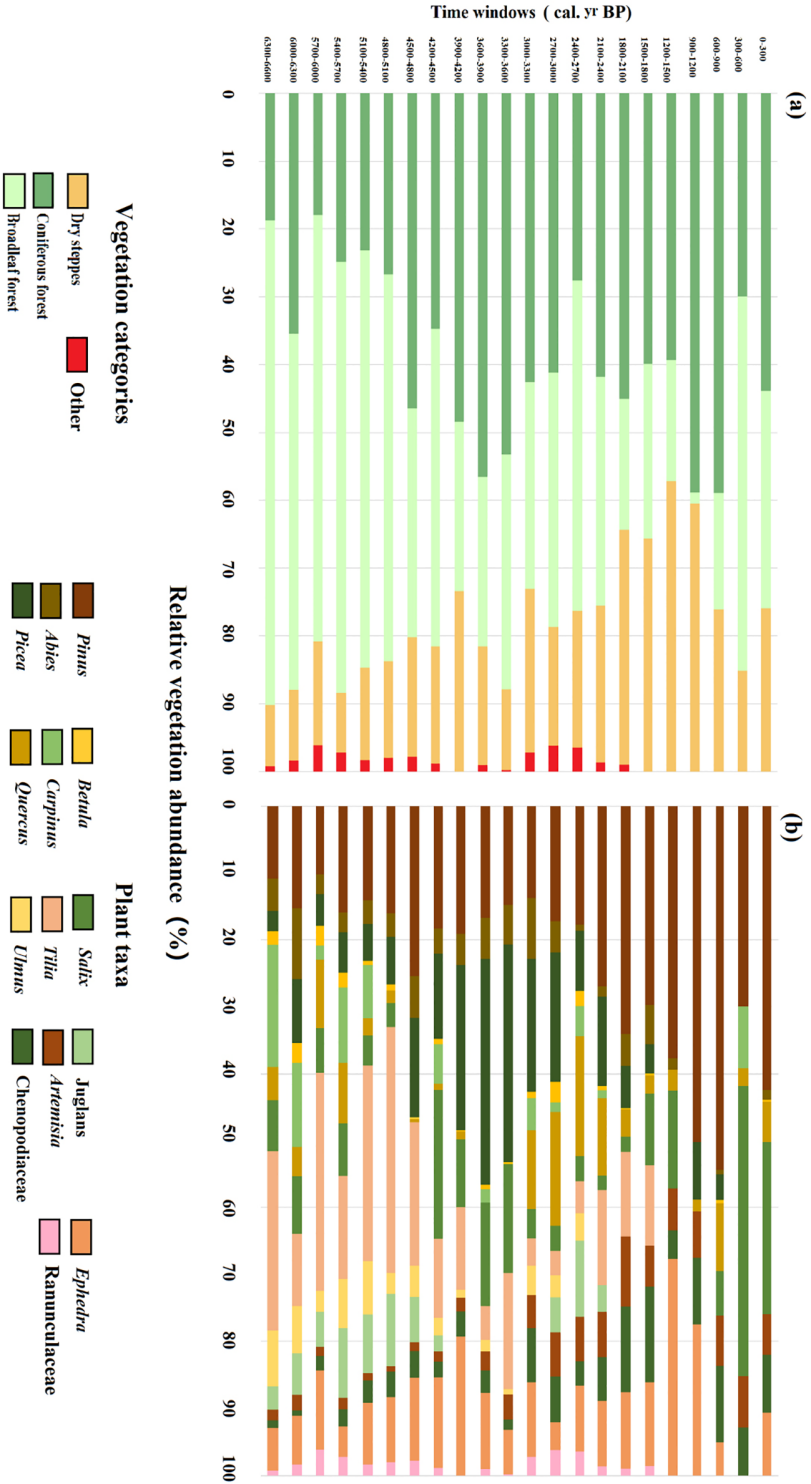
Vegetation reconstructions based on REVEALS using S4 with a wind speed of 3 m/s for marginal region. The four vegetation categories **(A)** and individual plant taxa **(B)** are shown.

Since the mid-Holocene, vegetation in the study region has consisted of varying proportions of coniferous forest, broadleaved forest, and dry steppe (**Figure 4**). Broadleaf forest dominated the area from 6600 to 4800 cal. yr BP, with a mean cover of 61.4%. *Tilia* was the dominant plant taxa in the broadleaved forest with a mean cover of 25.3%. On average, coniferous forest and dry steppe covered 24.5% and 11.9% of the region, respectively, with *Pinus* (mean cover 13.7%) the dominant conifer and *Ephedra* (mean cover 8.1%) the dominant dry steppe vegetation. The period 4800–2700 cal. yr BP was characterized by a reduction of the broadleaved forest and the relative increase especially in coniferous forest cover but also in the extent of dry steppe. *Tilia* cover decreased significantly while *Ulmus* and Juglans gradually decreased; *Picea* increased abruptly and replaced *Pinus* becoming the dominant plant taxa of the coniferous forest. From 2700 to 900 cal. yr BP, the extent of dry steppe further increased to reach its highest value 42.8% around 1200 cal. yr BP owing to the significant increase of *Ephedra* (mean cover 16.3%) and Chenopodiaceae (mean cover 8.5%). In the same period, the extent of coniferous forest varied but did not increase further, except within the period 1200–900 cal. yr BP when coniferous forest reached its highest value. *Pinus* (mean cover 32.6%) dominated the coniferous forest over approximately the last 2100 cal. yr BP whereas *Abies* and *Picea* were previously quite abundant as well. Broadleaved forest decreased to very low coverage (1.7%) between 1200 and 900 cal. yr BP. After 900 cal. yr BP, the cover of broadleaved forest increased again due to the increase of *Salix* (mean cover 25.2%)

### 4.3 Variation partitioning analyses

**Figure 5:**
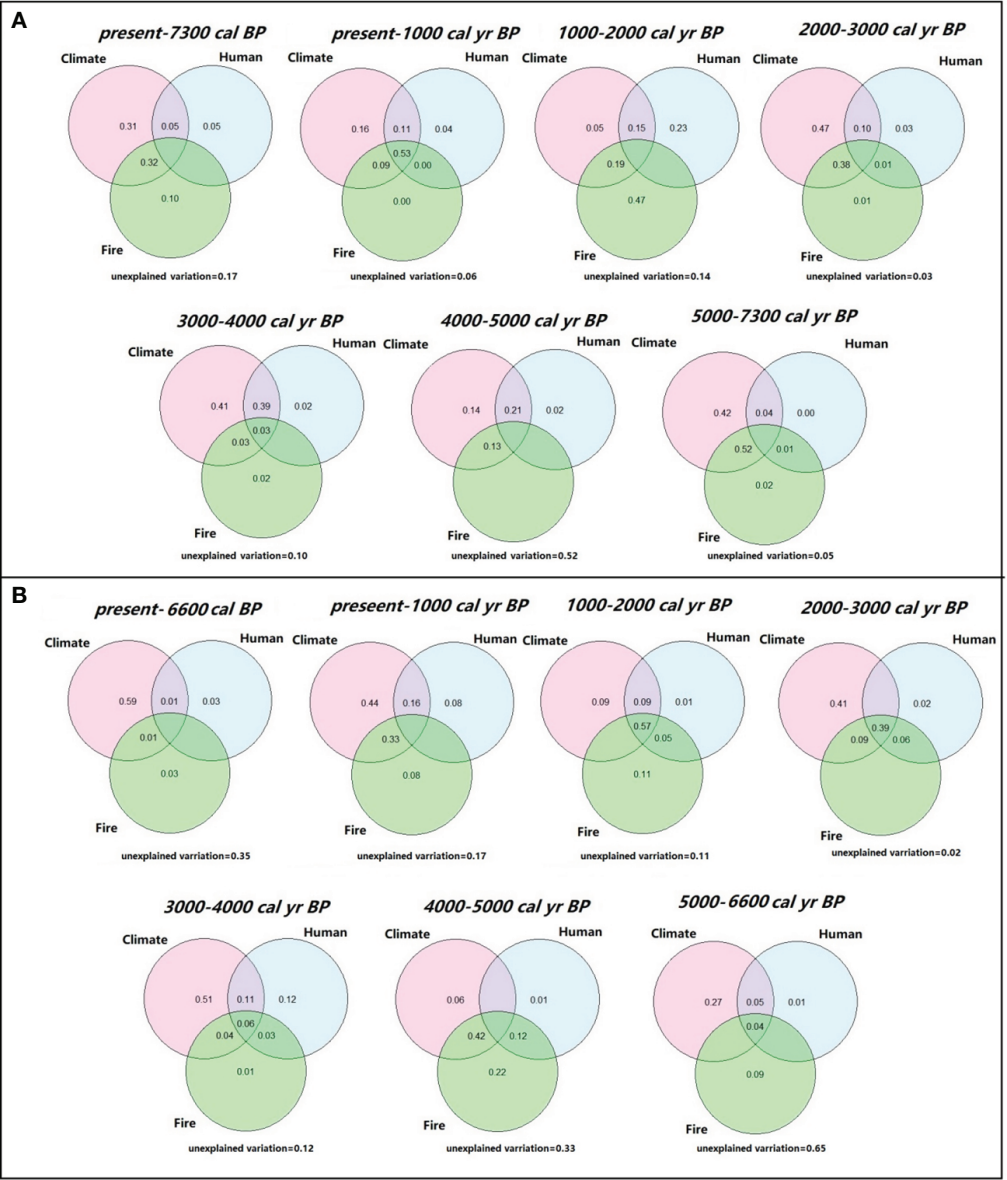
Variation in vegetation cover for central region **(A)** and marginal region **(B)** explained by climate, fire, and human activity during the whole study period and each time windows. Values below zero are not shown in the figures.

Variation partitioning analyses indicate that climate alone explained the highest fraction of variation (31%) in REVEALS estimated vegetation cover of the central region during the entire studies period (**Figure 5A**). The variation explained by human population size and fires, moreover, is lower (5% and 10%, respectively). Results of the moving window approach demonstrate that the influencing factors showed a variety of interaction patterns in different periods. During 7300–5000 cal. yr BP, the climate changes and fire frequencies together explained most of the vegetation cover changes followed by climate alone and relatively low human influences. During 5000–4000 cal. yr BP, climate changes and human population size together play the most important role in explaining 21% of the vegetation changes. During 4000–2000 cal. yr BP, climate changes explained most of the vegetation cover changes while fire frequencies and human population size also alliteratively make their contributions. During 2000–1000 cal. yr BP, the relative importance of fire frequency was significantly higher than that of other periods with 47% of the variation explained followed by human population size with 23% of the variation explained. After 1000 cal yr BP, climate changes, fire frequencies, and human population size together explained 53% of the vegetation cover changes.

When considering the full studied period for marginal region, climate alone also contributed the largest portion of variation (59%) in REVEALS estimates, while the contributions of human activity and fire frequency were very modest (**Figure 5B**). During 6600–5000 cal. yr BP, climate explained the highest variation of the vegetation cover (27%) followed by fire frequency and human activity explained 9% and 1%, respectively. During 5000–4000 cal. yr BP, climate changes and fire frequency together play the most significant role in explaining 42% of the vegetation changes. With the exception of the period between 1000 and 2000 cal. yr BP, when climate, fire frequency, and human activity collectively accounted for 57% of vegetation cover changes, climate changes had a significant impact on vegetation cover changes from 4000 cal. yr BP to the present, ranging from 41 to 51%.

## 5 Discussion

### 5.1 Influence of RPPs/FSPs/wind speeds on REVEALS estimates

RPPs, FSPs, and wind speeds are three critical parameters that influence the REVEALS outcomes. FSPs are calculated using Stoke’s law (Gregory, 1973) and the size of the pollen grains. FSPs can influence both REVEALS performance and the calculation of RPPs. Previous studies have shown that FSPs have generally a minor influence on the calculation of RPPs and REVEALS results because their values do not differ much from one pollen type to another compared to the large range in RPP values (e.g., Li et al., 2017; Zhang et al., 2017; Li et al., 2018). However, the choice of RPPs can significantly impact the results. The present calculations of RPPs were mainly based on modern pollen datasets, related FSPs, and corresponding vegetation data using the Extended R-value (ERV) model (Parsons & Prentice, 1981; Prentice & Parsons, 1983). Many factors, e.g., methodological and environmental factors, can influence the RPP calculations. For this reason, it is generally preferable to use a mean of various RPPs for the REVEALS applications (e.g., Mazier et al., 2012; Trondman et al., 2015), and therefore choosing the S4 alternative appears to be a good compromise among all available RPPs. However, to better understand the role of the RPPs on the REVEALS estimates, various alternatives were tested.

S1 was used by Li et al. (2020) to reconstruct Holocene land-cover change throughout China, whereas Zhang et al. (2021b) used S2 to reconstruct Holocene vegetation changes in the transition zone between subtropical and temperate ecosystems in east-central China. Compared to S2, S1 has fewer tree pollen types, especially for *Picea, Abies*, and *Salix*, which are common in marginal region through time. In contrast, S1 has more herb pollen types, such as Compositae, which are abundant in central region over time. For the other pollen types shared between the two alternatives, the RPPs/FSPs values are of the same order.

The RPPs of Cyperaceae and *Picea* in S3 proposed by Wieczorek & Herzschuh (2020) were much higher than their values in S1 and S2; note that Cyperaceae was excluded from all REVEALS runs because it is mainly derived from local vegetation in the study region and thus could add uncertainties in the REVEALS reconstructions. In marginal region, the cover of coniferous forest estimated by REVEALS was much lower using S3 compared with S2 because of the higher RRP values of *Picea* (See **Supplementary Material S2**-**Figure S3**).

Combining the data from S1, S2, and S3 for use in S4 generated a novel data set that includes most plant taxa that characterize the major vegetation types for both central and marginal regions. The REVEALS results using S4 agree with the cover of these vegetation types as represented on the vegetation map for the both central and marginal regions at a radius of 50 and 20 km, respectively (**Figures 3**, **4**). Therefore, as expected, RPPs and FSPs play a significant role in accounting for the discrepancies among the REVEALS estimates derived from the four alternatives.

Previous studies have reported that changes in wind velocity result in notable effects on pollen dispersal and deposition characteristics (Xu et al., 2016). Therefore, wind speeds might also have an effect on the REVEALS outcomes (See **Supplementary Material S2**-**Figures S4**, **5**). Our results show that the influence of wind speeds on REVEALS estimates differ among pollen types. For example, reconstruction of *Abies* cover was easily influenced by wind speeds because it is assumed that its heavy pollen grains cannot be transported as far under a wind speed of 3m/s compared to a wind speed of 6 m/s (Zhang et al., 2021b). The present study tested 3 m/s and 6 m/s wind speeds to explore their influence on the REVEALS results based on pollen records, and we found that wind speed of 6 m/s is suitable for central region while 3 m/s wind speed is more appropriate for marginal region. This result, moreover, aligns with present conditions in which central region is dominated by herbs and *Abies* is almost absent, whereas marginal region is dominated by trees and *Abies* is present.

### 5.2 Influence of basin type and size on REVEALS estimates

The present study uses pollen records from one large lake and two peatlands from large basins for the REVEALS model. Previous studies have shown that acquiring the most reliable estimates of regional vegetation cover using the REVEALS model should use pollen data sets from one or multiple large lakes with a water surface larger than 50 ha (Sugita, 2007; Hellman et al., 2008a). These underlying assumptions limit the application of the model because in many regions small-sized bogs and mires are generally more common than large lakes (Birgitte and Sugita., 2005; Mazier et al., 2012). To extend the use of the REVEALS model, some studies have examined whether data from small sites can be used for REVEALS modelling to accurately reconstruct the regional vegetation cover, and results have been positive (e.g., Trondman et al., 2016), although using small sites generate larger error estimates than when using large lakes. If the studied region does not include large lakes, using pollen samples from small basins (lakes and/or bogs) is recommended (Trondman et al., 2016). The use of large bogs, however, could add additional uncertainties, such as an overestimation of the local vegetation components. This could explain some of the differences observed between REVEALS reconstructions and the vegetation maps in the present study for the first time window.

When using pollen records from other basin types (e.g., marshes and peats) than lakes for REVEALS runs, the REVEALS assumption that there are no plants growing in the water body is violated (Sugita, 2007). In the case of bogs and peats, REVEALS has a specific algorithm to take this into account. However, this could be an issue for lakes where aquatic plants grow on the water surface. Pollen generated from those aquatic plants may bias the REVEALS reconstructions. To reduce this bias, pollen types generated from the local plants should be excluded before the REVEALS model runs (Mazier et al., 2012). In the present case, fern spores, *Typha*, and Cyperaceae pollen grains were excluded from DBSP pollen records and Cyperaceae from SYP pollen records.

Basins are regarded as the sink of pollen originating from both local and regional vegetation (Sugita, 2010). A larger basin size can receive more pollen transported from further distances representing vegetation information in a larger source area of pollen while a small basin size can represent more local vegetation information (Nielsen and Sugita, 2005). For instance, large lakes would have a source area of pollen of about 50 km, while a surface sample from smaller bogs or mires can would represent a smaller spatial extend (Prentice, 1988). Therefore, except for large lakes, when using pollen records from other different basin sizes for REVEALS estimates, the spatial scale of the reconstructed vegetation needs to be evaluated. In the present study, the referenced evidence of the Z max distances was selected based on the basin size of DBSL, DBSP and SY. DBSL was considered as the large lake and possibly with a pollen source area of around 50–100 km while SY and DBSP peatlands were considered as medium size basin with a pollen source area of around 10–50 km (Zhang et al., 2021). Therefore, we decided to choose these four distances (10, 20, 50, and 100 km) for vegetation evaluation. By comparing the REVEALS results with the regional vegetation map with different radius, our results show that central region can represent the regional vegetation within a radius of 50 km around DBSL and marginal region merely a source area of pollen of about 20 km, indicating their REVEALS results can well reflect the regional vegetation cover around the sampling site.

### 5.3 Responses of grassland vegetation to the 4.2 ka BP and IRD events

The “4.2 ka BP” event is considered one of the major global extreme climate fluctuations to have occurred during the Holocene, and it is also considered the transition from the middle to the late Holocene (Mayewski et al., 2004; Staubwasser & Weiss, 2006; Walker et al., 2012; Finkenbinder et al., 2016). The 4.2 ka BP event was first identified by Weiss et al. (1993) as an abrupt increase in aridity in Mesopotamia. Recognition of that event caused widespread concern when it was suggested that it induced considerable land-cover degradation and might be linked to the collapse of ancient civilizations (deMenocal, 2001; Weiss & Bradley, 2001).

In northern China, previous studies based on multiple proxies (e.g., stalagmite δ^18^O, ostracode assemblages, and grain-size distributions) have revealed that the 4.2 ka BP event was mainly associated with a continuous drought period which spanned from 4800–3600 cal. yr BP together with a mean annual temperature decrease of up to 2 °C (Fang & Hou, 2011; Xiao et al., 2018; Tan et al., 2018; Tan et al., 2020). The vegetation in Songnen grasslands has shown different patterns of response to the 4.2 ka event in the central and marginal areas of the region.

At the beginning of the 4.2 ka BP event (during 4800–4500 cal. yr BP), the climate became cold and arid and it can be seen that the Poaceae cover increased abruptly contributing to the steppe expansion accompanied by the degradation of tree cover in the center of the Songnen grasslands (**Figure 3**). Meanwhile, in the marginal Songnen grasslands, dry steppe cover increased slightly owing to the increase of *Artemisia* cover. Greater cover of *Pinus* and *Abies* in the marginal area at that time represented the rapid expansion of the coniferous forest. Total cover of the broadleaved forest decreased abruptly owing to the decrease of the *Tilia* cover (**Figure 4**).

The driest climate of the region occurred during the 4500–3900 cal. yr BP interval. In the central part of the Songnen grasslands (**Figure 3**), dry steppe first expanded abruptly with an increased cover of Compositae, Chenopodiaceae, and *Ephedra* from 4500–4200 cal. yr BP. That cover then decreased somewhat because of the slight increase in Poaceae cover during 4200–3900 cal. yr BP. In the margin of the Songnen grasslands (**Figure 4**) from 4500–4200 cal. yr BP, the cover of dry steppe remained stable while broadleaved forests expanded due to the sharp increase of the drought-tolerant *Salix* and accompanying decrease in coniferous forest cover. Between 4200–3900 cal. yr BP, dry steppe cover increased significantly mainly because of expansion of *Ephedra.* Meanwhile, *Pinus* and *Picea* replaced *salix* while *Tilia* became the dominant tree taxa, leading to the expansion of coniferous forests and contraction of broadleaved forest cover.

At the end of the 4.2 ka BP event (during 3900–3600 cal. yr BP), the climate remained cool and dry. In central part of the Songnen grasslands, tree cover rose slightly with expansion of *Pinus* and *Picea* cover, while the dry steppe cover increased as the steppe cover decreased (**Figure 3**). In the marginal part of the Songnen grasslands, broadleaved forest cover remained the same as in the previous interval, a decrease in *Ephedra* led to a reduction in dry steppe, and the coniferous forest continuously expanded to a relatively high level (**Figure 4**).

**Figure 6:**
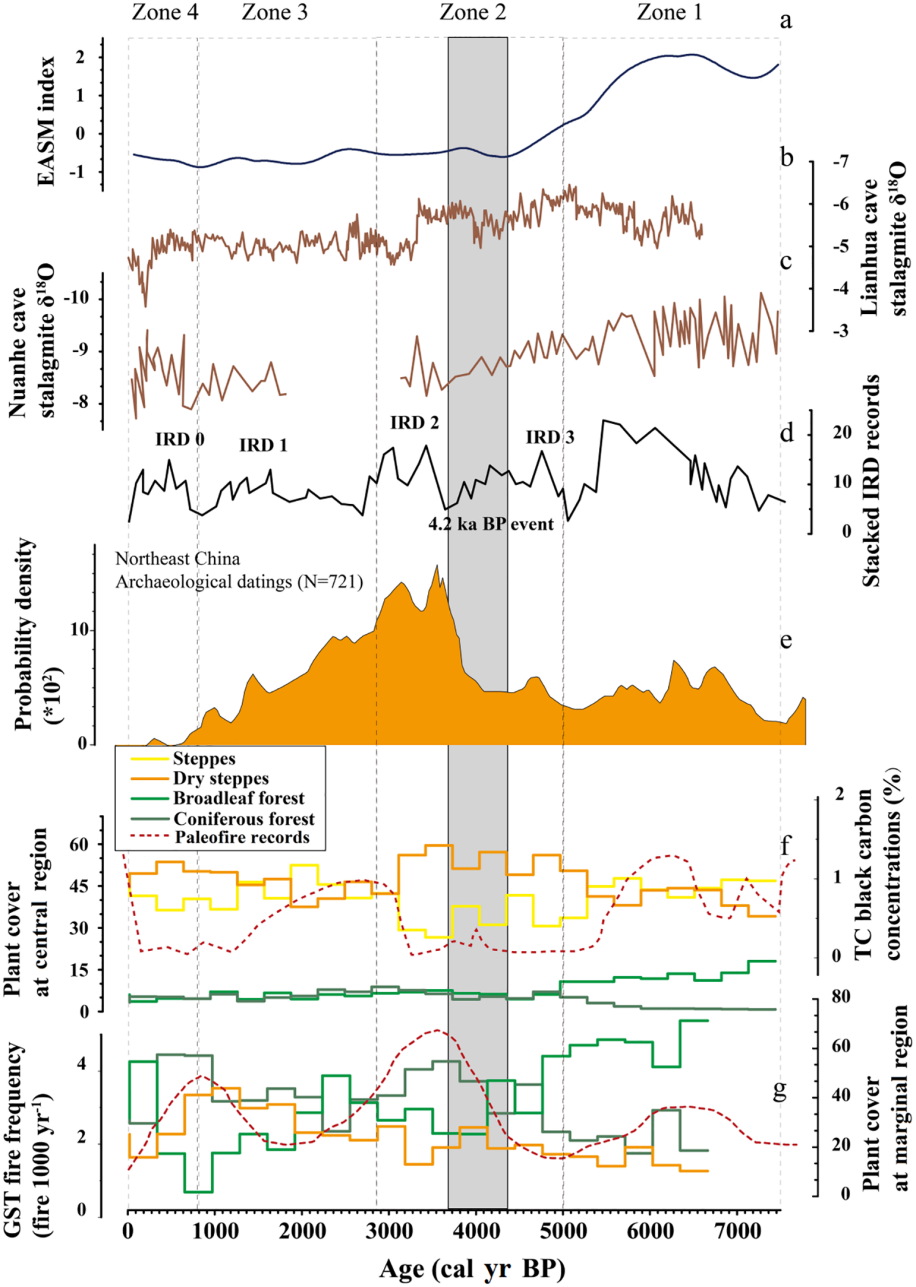
Comparisons of regional climate, fire and human activity records with Songnen grasslands vegetation changes since the mid-Holocene. **(A)** The EASM index is referenced from a published paper (Chen et al., 2008); **(B)** The stalagmite δ^18^O records are from Lianhua Cave, northern China (Cosford et al., 2009); **(C)** The stalagmite δ^18^O records are from Nuanhe Cave, northeastern China (Wu et al., 2011); **(D)** Holocene stacked IRD events in the North Atlantic (Bond et al., 2001); **(E)** Human activity records in northeastern China (Wang et al., 2020); **(F)** The REVEALS vegetation cover based on pollen records from central region, this study with black carbon records (Peng et al., 2021); **(G)** The REVEALS vegetation cover based on pollen records from marginal region, this study with fire frequency records (Meng et al., 2020).

In addition to the 4.2 ka BP event, the reconstructed vegetation changes in both the central and marginal parts of the Songnen grasslands were also closely associated with the stacked ice-rafted debris (IRD) events identified in the North Atlantic Ocean (Bond et al., 2001; **Figure 6**). After 4.2 ka BP, the regional climate became wetter, yet remained cool, in association with the IRD2 event. Dry steppe and coniferous forest continued to dominate the central and marginal Songnen grasslands, respectively, until approximately 2700 cal. yr BP. Although the cover of broadleaved forest remained stable, cold-tolerant *Betula* replaced *Salix* as the dominant taxa. From 1200 to 600 cal. yr BP, influenced by the IRD 0 event, dry steppe in the central Songnen grasslands re-expanded to a relatively high level while steppe and tree cover both decreased slightly. In the marginal grasslands, rapid increase in cover of *Pinus* and *Ephedra* contributed to expansion of coniferous forest and dry steppe, respectively, and contraction in the extent of broadleaved forest.

The response of regional vegetation to climate change consists of the response of individual species and how those species interact (Davis & Shaw, 2001). In general, vegetation of the Songnen grasslands, especially such drought-tolerant plants as *Ephedra*, Compositae, and *Salix*, respond rapidly to abrupt climate change. The taxa comprising a vegetation type, however, may adjust before the total cover of the vegetation type changes.

### 5.4 Role of climate, fire, and human activity in vegetation dynamics of Songnen grasslands

In this study, we quantify the relative importance of climate, fire, and human activity to mid-late Holocene vegetation dynamics for both central and marginal regions in Songnen grasslands and clearly demonstrated that climate was the dominant driving forces. In addition, compared to the human activity, fire frequency contribute more to vegetation dynamics, especially for central region. Our results answer the argument about whether human activity have dominated the grasslands vegetation changes after the mid-Holocene. In fact, we found that vegetation patterns are the result of long history of climate, fire, and human interactions and the impacts of human activities intensified especially for the last two millennia.

In Songnen grasslands, climate was influenced by EASM circulation variations that are mainly controlled by changes in solar insolation (Zhang et al., 2003; Berger et al., 2007). Relatively high solar insolation contributes to high surface temperatures of the western Pacific Ocean, which induces northward migration of the Western Pacific Subtropical High (WPSH). That migration strengthens the EASM, which leads to a precipitation increase in northeastern China (Zheng et al., 2018; Zhang et al., 2020). Likewise, relatively low insolation is associated with a precipitation decrease in this region. Previous work has reported that EASM-associated precipitation has been a major influence on vegetation changes in northern China (Chen et al., 2015; Stebich et al., 2015; Zhou et al., 2016). Therefore, we hypothesize that the EASM circulation and monsoon precipitation are the direct driving force for the vegetation dynamics in the Songnen grasslands since the mid-Holocene. Comparing REVEALS modeling results for the two Songnen grassland subregions with regional EASM variations has shown that these trends are generally consistent (**Figure 6**). Between 7500 and 5000 cal. yr BP (Zone 1), EASM circulation maintained the highest level as indicated by the low stalagmite δ^18^O value from both Nuanhe cave and Lianhua cave. More wet and warm air was brought by the strengthened EASM from the Pacific Ocean to northern China and therefore increased the precipitation. The warm and humid climate led to the dominance of broadleaved trees in the marginal areas and steppe in the central part of the Songnen grasslands. Before the 4.2 ka BP event (Zone 2), continued weakening of the EASM resulted in a reduction of the water vapor supply to northeastern China. The cooler and drier climate drove the expansion of dry steppe in the central area of Songnen grasslands, while the marginal area of grasslands experienced a gradual replacement of broadleaved forest with coniferous forest. After 4.2 ka BP until 1000 cal. yr BP (Zone 2 & Zone3), the EASM initially strengthened slightly then gradually weakened but generally maintained in a relatively low level (low value of EASM index) compared to the early mid-Holocene. In the same interval, precipitation fluctuated dramatically in response to variations of the EASM. Vegetation patterns in the Songnen grasslands responded rapidly to these climate changes. Broadleaved forest and coniferous forest alternately dominated the Songnen grasslands margin, while steppe and dry steppe alternately dominated the central Songnen grasslands. From 900 cal. yr BP to the present (Zone 4), the EASM strengthened slightly accompanied by a rise in the regional mean annual precipitation. Coniferous forest and dry steppe cover decreased significantly as broadleaved forest cover increased in the marginal Songnen grasslands, while steppe continued to expand and eventually replaced dry steppe to dominate the central Songnen grasslands.

The response patterns of vegetation to fire are complicated and generally site-specific (Seddon et al., 2015). In this study, fire acts as a catalyst for vegetation to adapt to climate changes by destroying the original vegetation stability with the help of resilience and resistance allowing the vegetation to respond rapidly to climate (Dar et al., 2020). For instance, fire contribute to the rapid transformation of steppe and dry steppe in central region, and transformation of coniferous forests and broadleaved forest in marginal region, respectively (**Figures 6F, G**). Moreover, the interaction pattern of vegetation and fire was different in the two regions. For central region dominated by herb vegetation, fire intensified during the strong EASM periods possibly related to the relatively high cover of biomass as the fuel burning (Tong et al., 2009). In contrast, for marginal region dominated by trees, fire intensified during the low EASM periods possibly because the expanded coniferous trees with relatively high flammability (Cohn et al., 2011).

For both central and marginal regions, the effects of human activity on vegetation dynamics were minimal. Our study’s findings diverge from those of earlier research conducted in northern China, where vegetation was found to be impacted by human-induced land-use change-related landscape opening and forest clearing (Liu et al., 2021). These differences could be the result of the mid-Holocene human activity patterns that varied by region. Ancient inhabitants frequently engaged in deforestation practices to expand their agricultural fields. Prior to historical times (7300–2000 cal. yr BP), inhabitants in our research area, however, consistently engaged in a typical fishing and hunting culture with little livestock rearing and little agricultural activity (Li, 2008). Even though the human population rose significantly after entering the Bronze Age around 4000 cal yr BP, this subsistence strategy only has a minor impact on the grassland vegetation cover. Continuously indigenous dynasties were established in the Changbai mountain regions after 2000 cal. yr BP, and cities with better carrying capacities were built, aiding in the population aggregation (Li, 2006; Jin & Xiao, 2016). As a result, the Songnen grasslands’ agricultural population shrank, and for a considerable amount of time, a small number of nomadic people ruled the area (Zhang, 2009).

## 6 Conclusions

Three pollen records from the Songnen grasslands were applied to the REVEALS model to quantitatively reconstruct the vegetation cover of the region during the middle-late Holocene. Output from the REVEALS scenarios revealed the significance of choosing appropriate model parameters such as RPPs, FSPs, and wind speeds on the vegetation cover estimates. The S4 alternatives, which used means of available RPPs and FSPs values, with wind speeds of 6 m/s and 3 m/s, were chosen to reconstruct quantitative plant cover in the central and marginal Songnen grasslands for the radial extent of 50 km and 20 km from the sampling sites, respectively. The higher wind speed alternatives appeared to work better in steppe areas (central region) compared to more forested landscapes (marginal region).

Based on the best modeling alternatives, results showed that the vegetation patterns have varied in the central and marginal areas of the Songnen grasslands since the mid-Holocene. Steppe and dry steppe were dominant in the central part of the Songnen grasslands, while the marginal grasslands were mainly characterized by broadleaved forest and coniferous forest.

The Songnen grasslands vegetation was sensitive to 4.2 ka BP and IRD events. The results showed that during these cooling events, taxa composition in a vegetation type changed faster than the total cover of the vegetation type because of climate sensitivities of the drought-tolerant plants.

Since the mid-Holocene, changes in EASM and the associated influences on precipitation have mostly been responsible for the vegetation change in the Songnen grasslands. Fire also had a significant part in helping the vegetation adapt to the changing environment while human activities only had minor effects. These findings should help in understanding how vegetation may change in the Songnen grasslands in the future under the current global warming trends.

## Data availability statement

The original contributions presented in the study are included in the article/**Supplementary Material**. Further inquiries can be directed to the corresponding author.

## Author contributions

HN: Conceptualization, Methodology, Investigation, Writing- Original draft preparation; LM: Writing - Review and Editing; DS: Writing - Review and English Editing; GG: Data curation; JW: Visualization; MM: Formal analysis; Linlin Liu: Formal analysis; Lina Song: Conceptualization; DJ: Funding acquisition. All authors contributed to the article and approved the submitted version.
