# Supplementary material for: Middle to late Holocene plant cover variation in relation to climate, fire, and human activity in the Songnen grasslands of northeastern China
Source: Front Plant Sci. 2023 Jan 9;13:1071273. doi: 10.3389/fpls.2022.1071273 (PMC9868567; doi:10.3389/fpls.2022.1071273)
Supplement: Supplementary file 1 [file DataSheet_1.docx]

**Supplementary Material S2**

**Middle to late Holocene plant cover variation in relation to climate, fire, and human activity in the Songnen grasslands of northeastern China**

This file includes:

**Supplementary Texts**

**Text 1:** The detailed ArcGIS procedure to extract the vegetation information

**Text 2:** The detailed results of REVEALS estimates versus regional vegetation maps

**Supplementary Figures**

**Figure S1**. REVEALS estimates based on different scenarios of Zmax and wind speeds for the central region.

**Figure S2.** REVEALS estimates by using different scenarios of Zmax and wind speeds for the marginal region.

**Figure S3.** Comparison between the different REVEALS alternatives for mean coniferous plant cover and coniferous forest cover for the marginal region.

**Figure S4.** Different REVEALS scenarios for the Central region and the first-time window.

**Figure S5.** Different REVEALS scenarios for the Marginal region and the first-time window.

**Figure S6.** Lithology, ‘Bacon’ age–depth models and accumulation rate plot for DBSP core.

**Figure S7.** Lithology, ‘Bacon’ age–depth models and accumulation rate plot for SYP core.

**Figure S8.** Lithology, ‘Bacon’ age–depth models and accumulation rate plot for DBSL core.

**Figure S9.** Pollen percentages diagram of SYP core (A) and REVEALS-based vegetation reconstructions (B) for the marginal region.

**Figure S10.** Pollen percentages diagram of DBSL (A) and DBSP (B) cores and REVEALS-based vegetation reconstructions (C) for the central region.

**Figure S11.** Land cover for the three vegetation categories based on the 10, 20, 50, and 100 km radius maps for the central region versus REVEALS estimates.

**Figure S12.** Land cover for the three vegetation categories based on the 10, 20, 50, and 100 km radius maps for the marginal region versus REVEALS estimates.

**Supplementary Texts**

**Text.1. Procedure to extract the vegetation information using ArcGIS**

After delimiting the study area with the “clip” function, the “symbology” function was used to categorize unique values for each vegetation type. The “count VGTCLASS value” was then used to acquire the relative vegetation cover for the whole area, and for specific vegetation types in a known radius of the study area.

**Text.2. REVEALS results versus regional vegetation maps**

1. *Central region*

The use of various Zmax values ranging from 10 to 100 km had little influence on the REVEALS outcomes for the central region (Fig. S1), although the cover of dry steppe decreased slightly and steppe cover increased with increasing Zmax. Therefore, for comparing the vegetation maps, we considered only the REVEALS runs for a Zmax of 50 km, which is assumed to be a good representative and compromise value among all the Zmax values.

According to the vegetation maps, for the central region, increasing the radius from 10 km to 50 km led to the gradual decrease in cover of dry steppe from more than 50% to approximately 35% (Fig. S11). Meanwhile, steppe and broadleaved forests both showed a significant increase, from 11% to 36% and 0 to 11%, respectively. For the radius of 100 km, the cover of broadleaved forests changed little compared to that within the radius of 50 km, while the dry steppe cover increased, reaching the same cover as steppe.

The REVEALS runs using wind speeds of 3 m/s generally overestimated the steppe cover, in particular for options S2 and S3 (Fig. S11) for which the steppe cover reached ca. 55% and 60%, respectively. For S1 and S4, steppe cover was near 50%. The coverage amount for dry steppe followed an opposite trend with REVEALS slightly overestimating dry steppe coverage when using S1 and S4 options, and underestimating it when using S3. When using S2, REVEALS provided a value for dry steppe similar to that from the map. Broadleaved forests are generally underestimated by REVEALS, except for with S3.

When using a wind speed of 6 m/s, the cover of dry steppe increased for all four RPP alternatives compared to its estimate in the 3 m/s option (Fig. S11). More specifically, for S1, the cover of dry steppe nearly reached 50%, which is greater than the cover of steppe. In general, the REVEALS estimates for steppe and dry steppe combined were close to what they were when using the 3 m/s option. The proportions of broadleaved forests were influenced little by the change in wind speeds for the model runs.

The REVEALS results showed an overestimation in steppe cover under 3 m/s wind speed conditions compared with the vegetation map at a radius of 50 km. However, with 6 m/s wind speeds, REVEALS estimates were closer to the results calculated from maps with the radius of 50 km for S2, S3, and S4, in particular.

1. *Marginal region*

The use of various Zmax radius values had little influence on the REVEALS outcomes. The cover of dry steppe decreased with increasing Z max while the cover of coniferous forests increased (Fig. S2). When comparing with the vegetation maps, only the REVEALS runs for a Zmax of 50 km was considered, as was the case for marginal region. Employing a range of RPP alternatives and two wind speeds (Fig. S12) influenced the REVEALS outcomes.

Based on calculations from the vegetation map for marginal region, the cover of the three vegetation types changed significantly with variations in the radial distance (Fig. S12). Broadleaved forest reached 10% within the radius of 10 km, while coniferous forests and dry steppes were absent. With a radius of 20 km, the highest cover of coniferous forests are seen at approximately 38%, and this is much higher than the broadleaved forest (23%) and dry steppe cover (14%). Broadleaved forest cover increased to approximately 40% and 35% and coniferous forest cover fell to about 28% and 24% when the radius was enlarged to 50 km and 100 km, respectively. Dry steppe cover remained at about 10% in both cases.

The REVEALS outcomes using a wind speed of 3 m/s showed a general overestimation of coniferous forest. That overestimation was particularly high for scenarios S1 and S2, which generated coniferous forest covers much higher (exceeding 50% and 60%, respectively) than the vegetation maps for radius values of 20**–**100 km. The proportion of dry steppe was also overestimated by REVEALS. Broadleaved forest was underestimated by REVEALS for S1, S2, and S4 but were similar to the map for S3.

When a wind speed of 6 m/s was used in REVEALS (Fig. S12), the model’s overestimation of coniferous forest was smaller than for a wind speed of 3 m/s; the REVEALS values were closer to the ones derived from the 20**–**100 km map. The extent of broadleaved forest was still underestimated by REVEALS, especially for the S1 option. In contrast, a wind speed of 6 m/s the model produced a greater overestimate of dry steppe than it did using a wind speed of 3 m/s for all RPP scenarios.

In general, compared to the vegetation map, within the radius of 20**–**100 km REVEALS overestimated the cover of dry steppe at a wind speed of 6 m/s. When a wind speed of 3 m/s was used, REVEALS estimates were closer to the results calculated from the 20 km map for S4, in particular; however, all vegetation categories are slightly overestimated.

**Supplementary Figures**


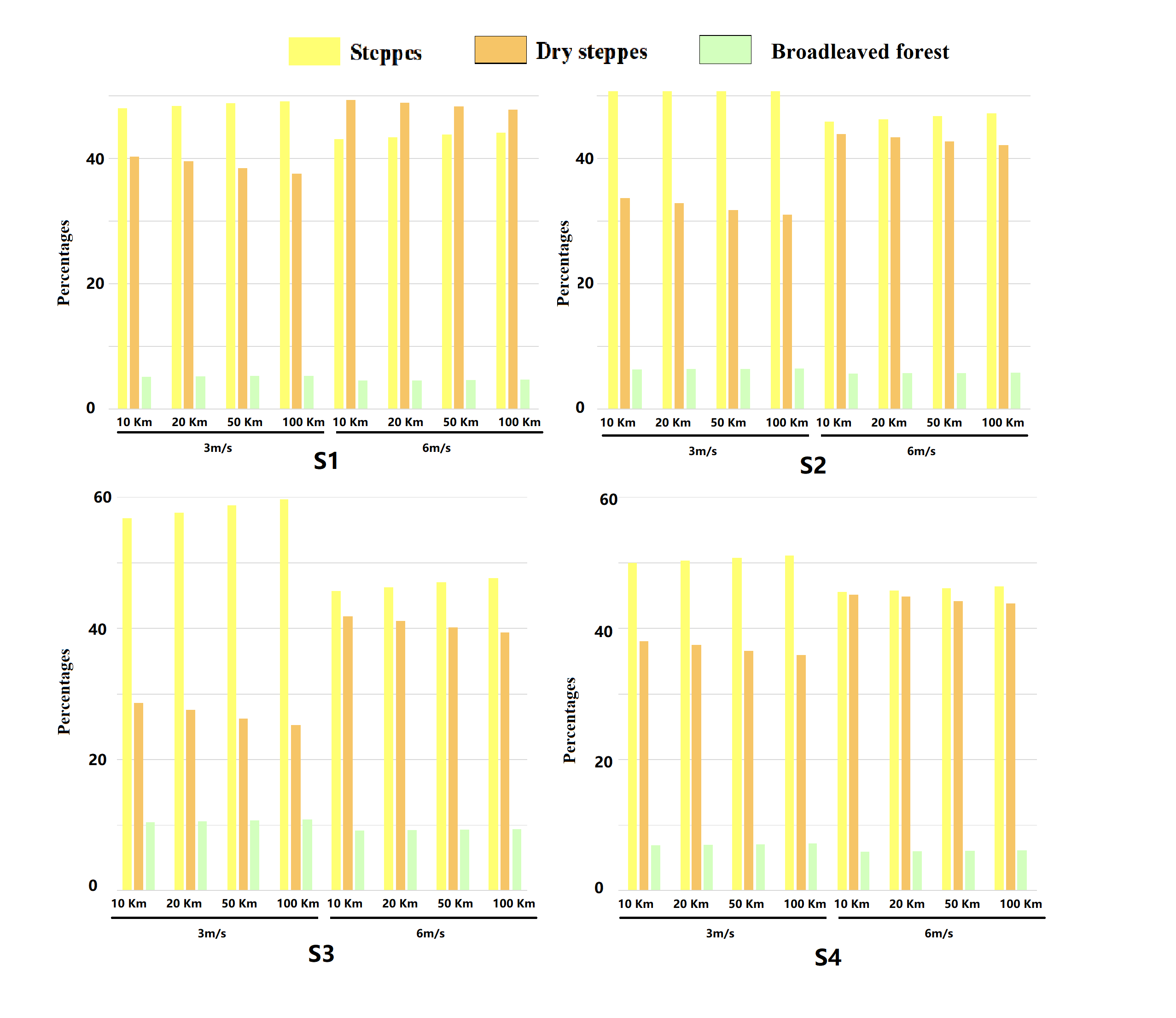


**Fig. S1.** REVEALS estimates based on different scenarios of Zmax and wind speeds. The Central region is shown here. The different vegetation categories are expressed in percentage of the total land cover.


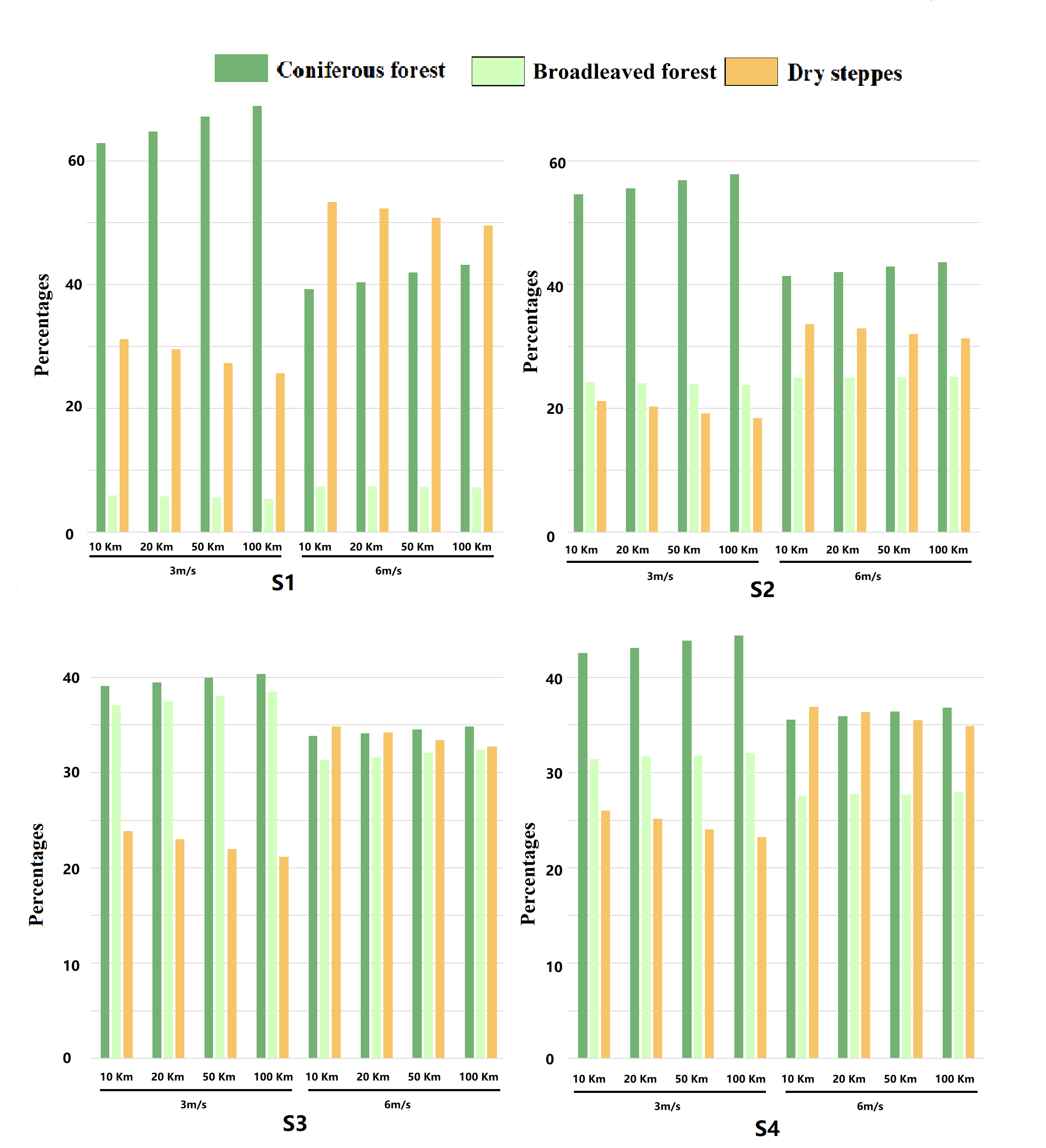


**Fig. S2.** REVEALS estimates by using different scenarios of Zmax and wind speeds. The Marginal region is shown here. The different vegetation categories are expressed in percentage of the total land cover.


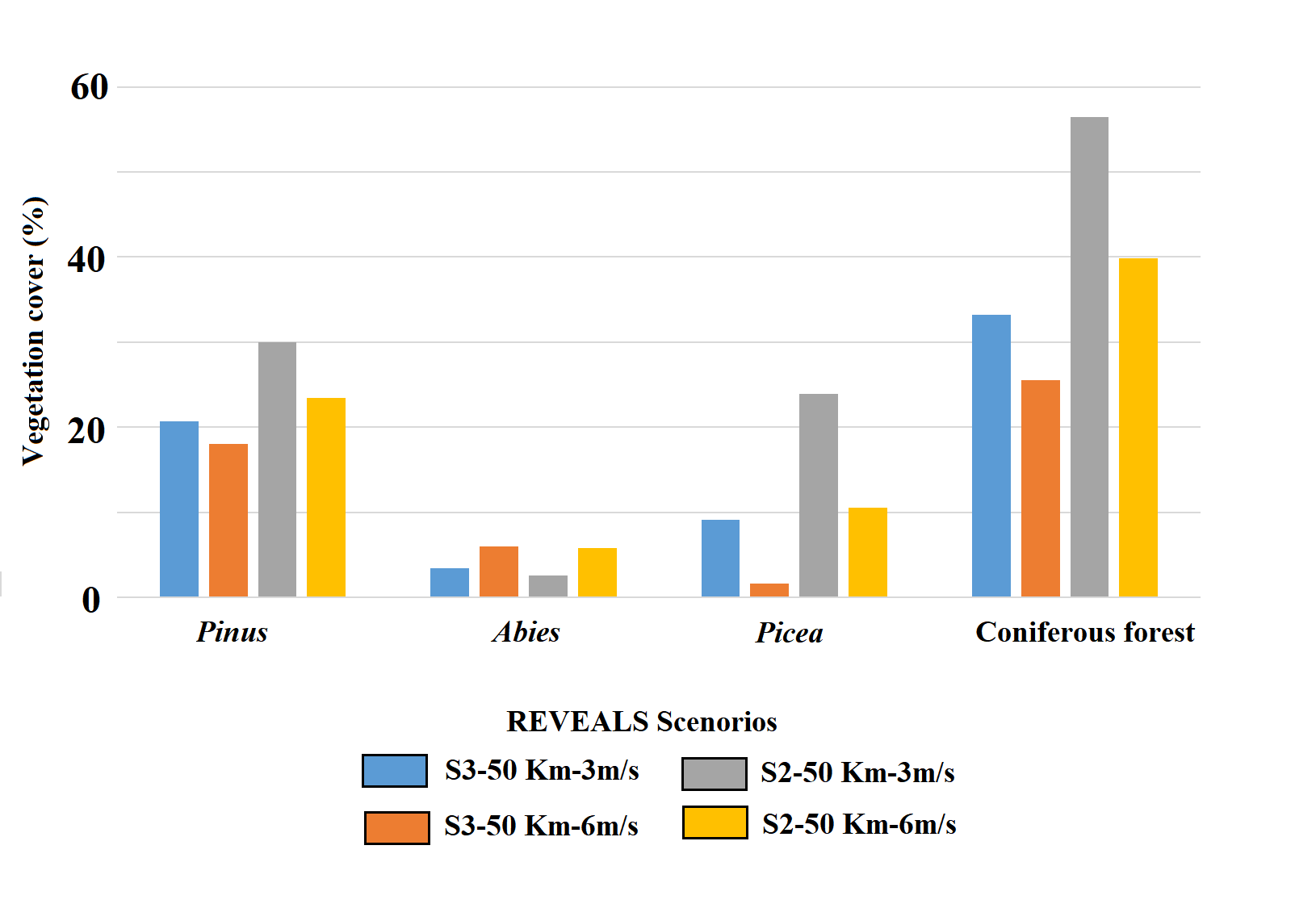


**Fig. S3.** Comparisons of using different REVEALS alternatives for estimating the mean coniferous plant cover and coniferous forest cover for the marginal region.


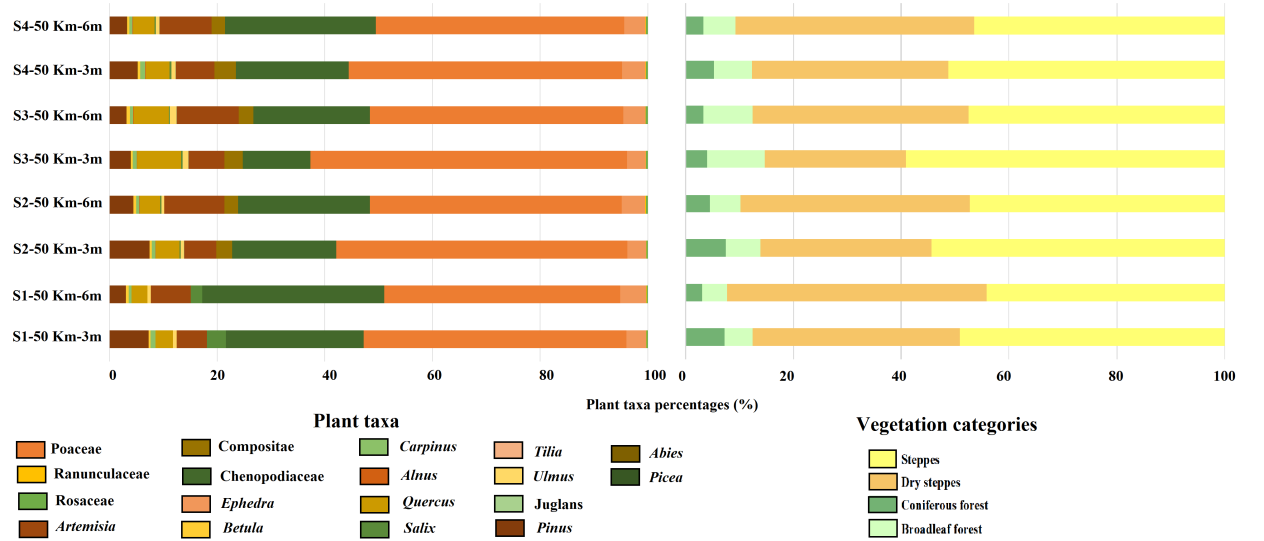


**Fig. S4.** Central region: REVEALS estimates based on different scenarios and the first-time window.


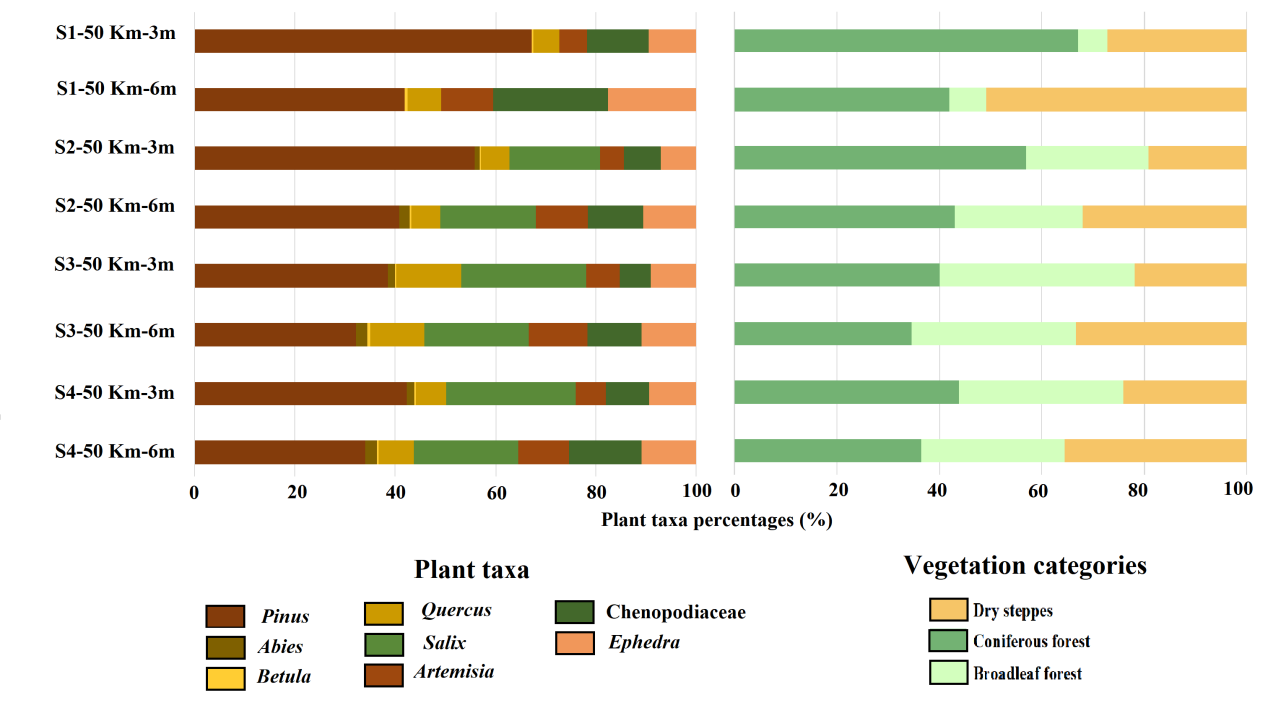


**Fig. S5.** Marginal region: REVEALS estimates based on different scenarios and the first-time window.


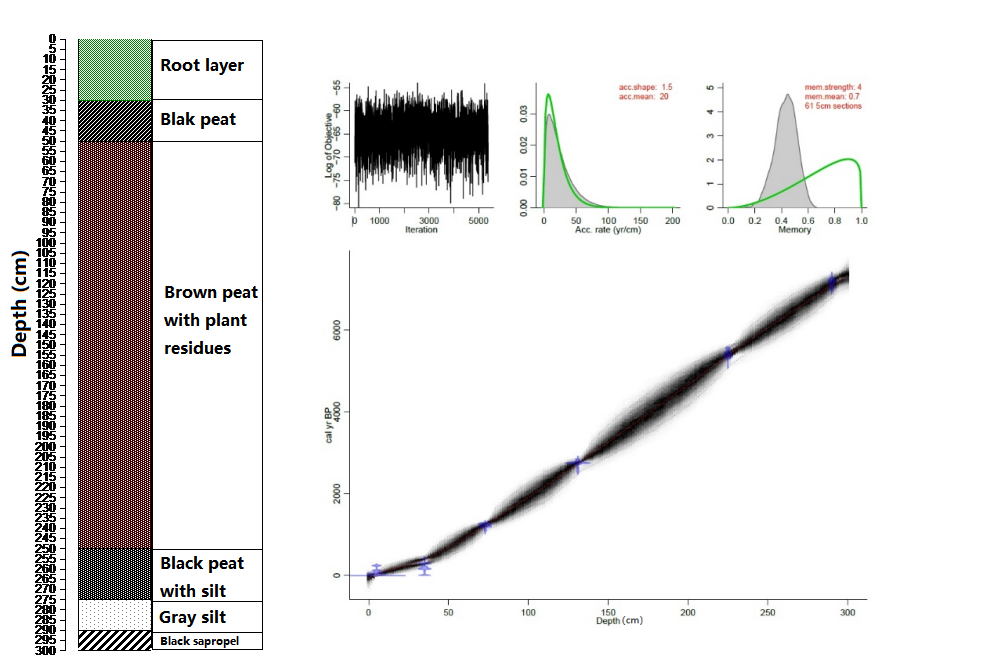


**Fig. S6.** Lithology, ‘Bacon’ age–depth models and accumulation rate plot for DBSP core.


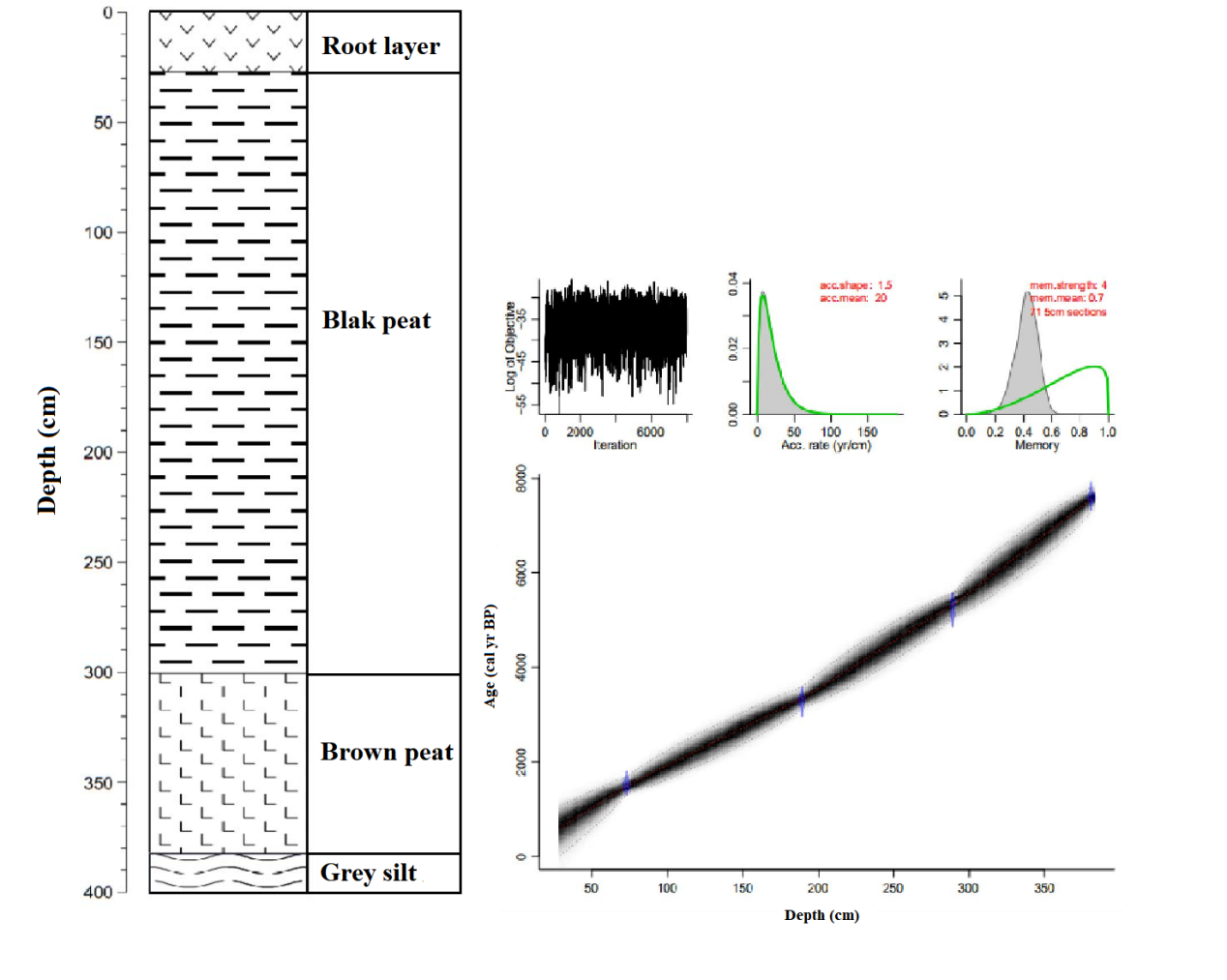


**Fig. S7.** Lithology, ‘Bacon’ age–depth models and accumulation rate plot for SYP core.


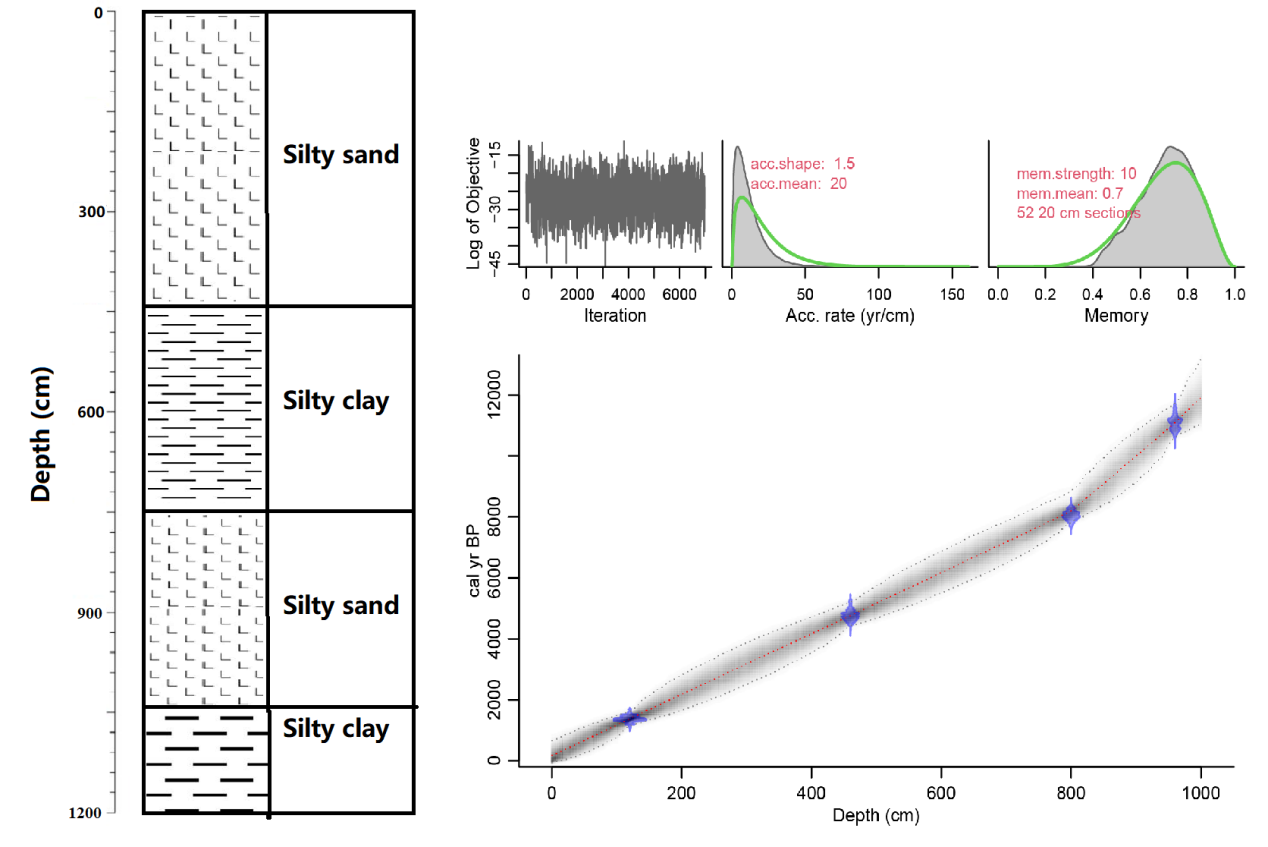


**Fig. S8.** Lithology, ‘Bacon’ age–depth models and accumulation rate plot for DBSL core.


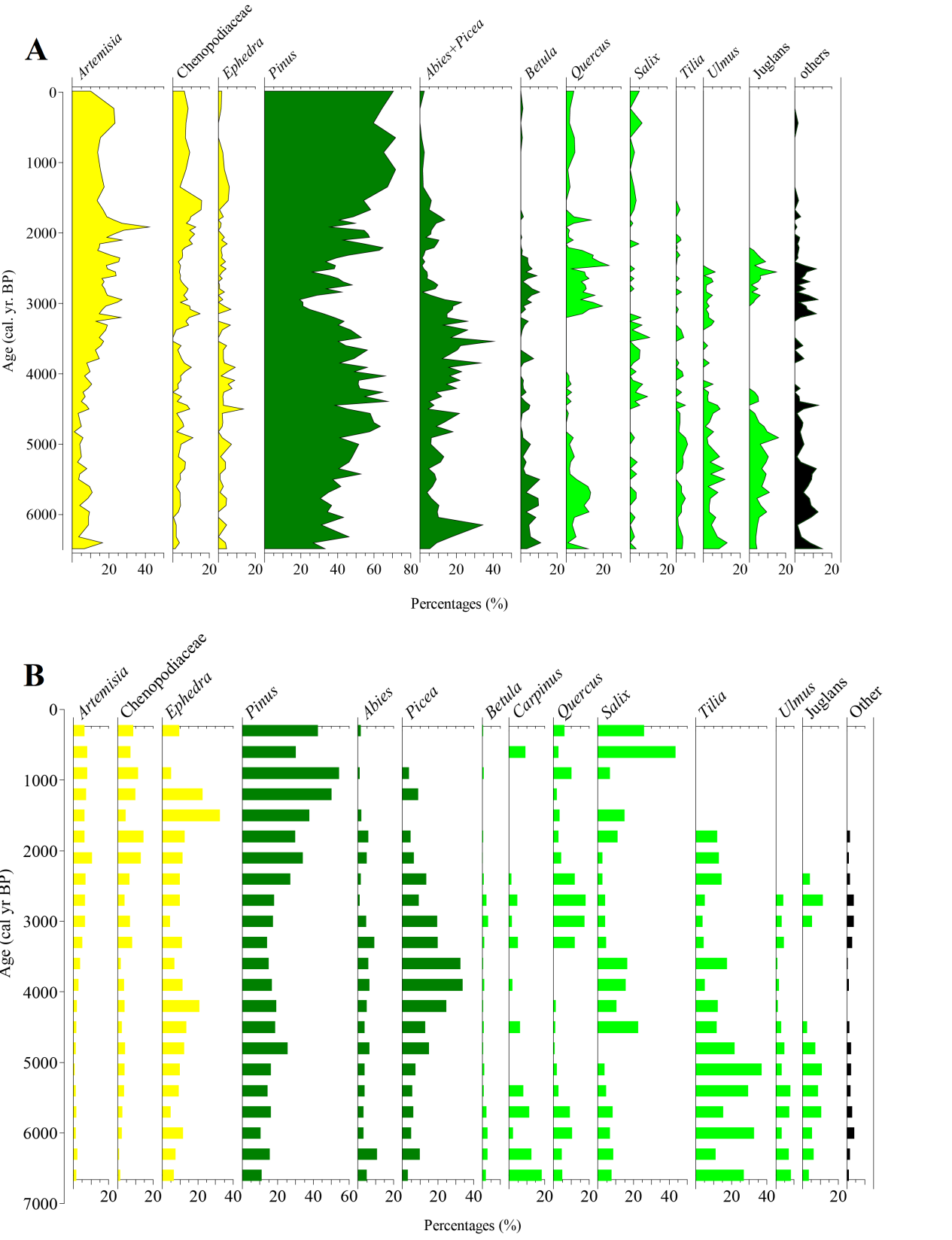


**Fig. S9. Marginal region:** Pollen percentages diagram of SYP core (A) and REVEALS-based vegetation reconstructions (B). The pollen record from the marginal region is characterized by a large amount of broadleaved and conifer tree pollen types, representing approximately 70% of the entire SYP record.


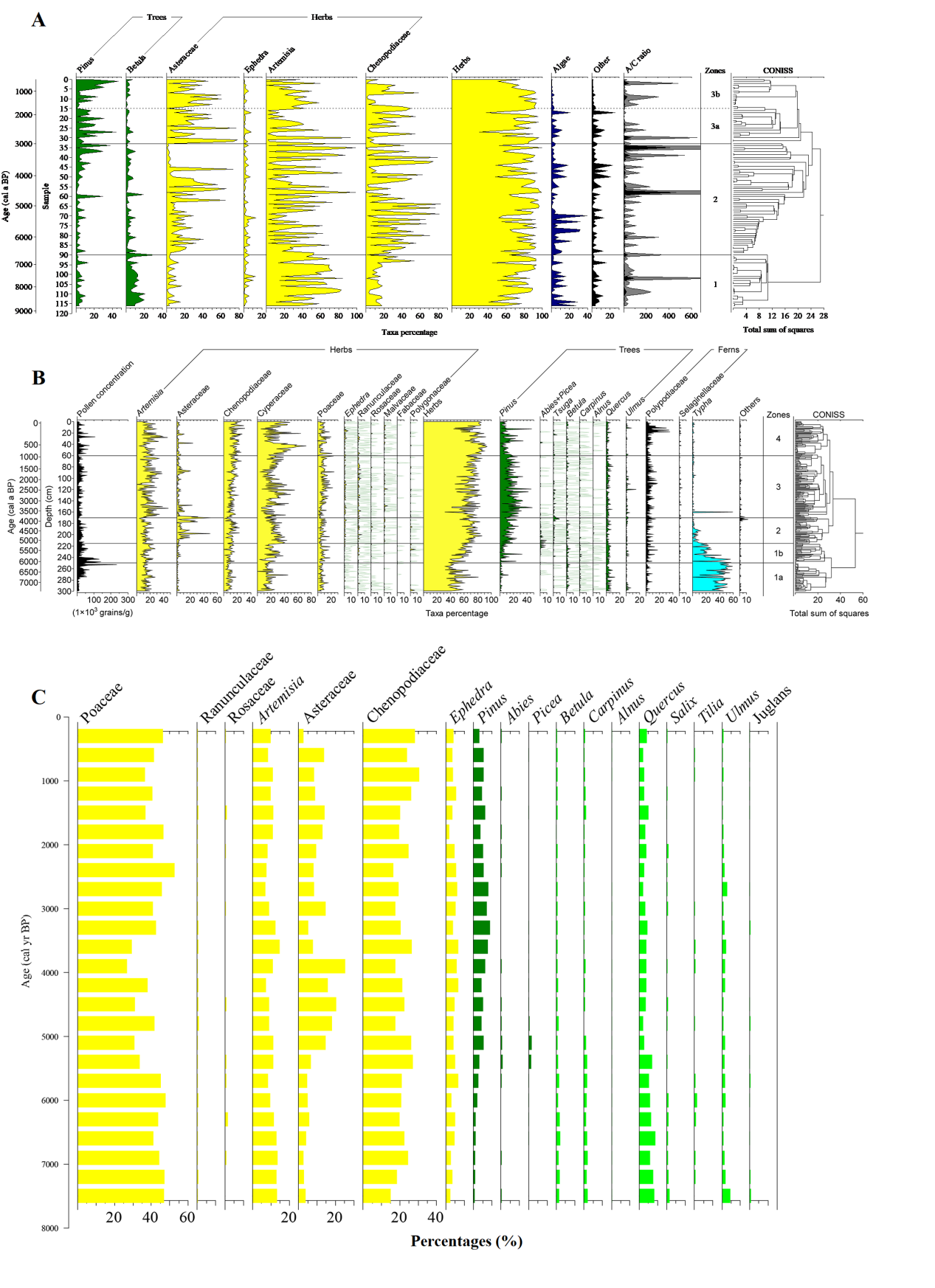


**Fig. S10. Central region:** Pollen percentages diagram of DBSL (A) and DBSP (B) cores and REVEALS-based vegetation reconstructions (C). The pollen records from the central region are mainly characterized by herbs; herbaceous pollen accounts for 77.8% and 65.9% for DBSL and DBSP, respectively.


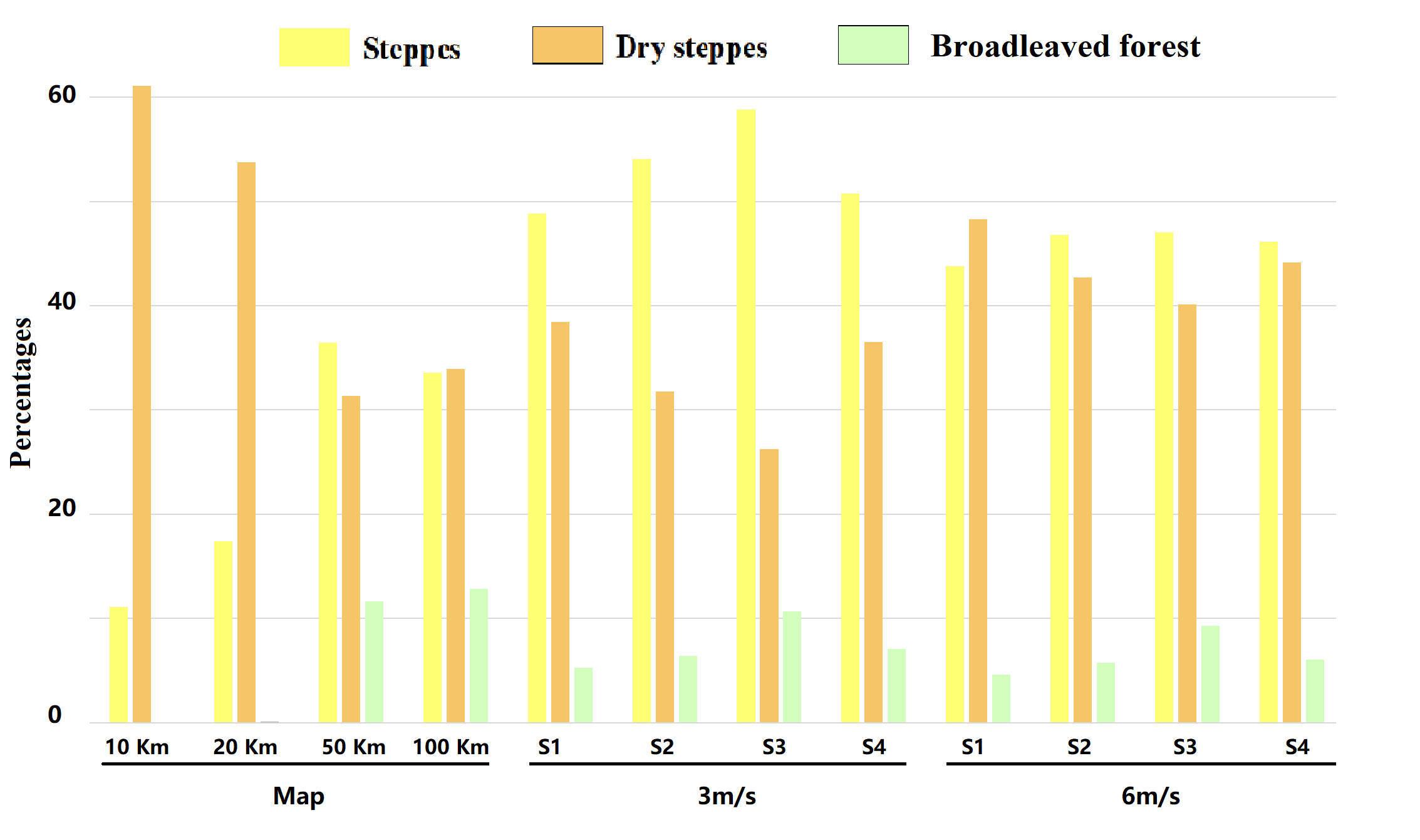


**Fig. S11. Central region:** Land cover of the three vegetation categories based on the 10, 20, 50, and 100 km radius maps versus REVEALS estimates. All REVEALS reconstructions correspond to a Zmax of 50 km. S1, S2, S3, and S4 are related to different RPP options used for the model runs. The two wind speed alternatives are also shown, i.e., 3 and 6 m/s. See the Methods section for more explanation about the model settings.


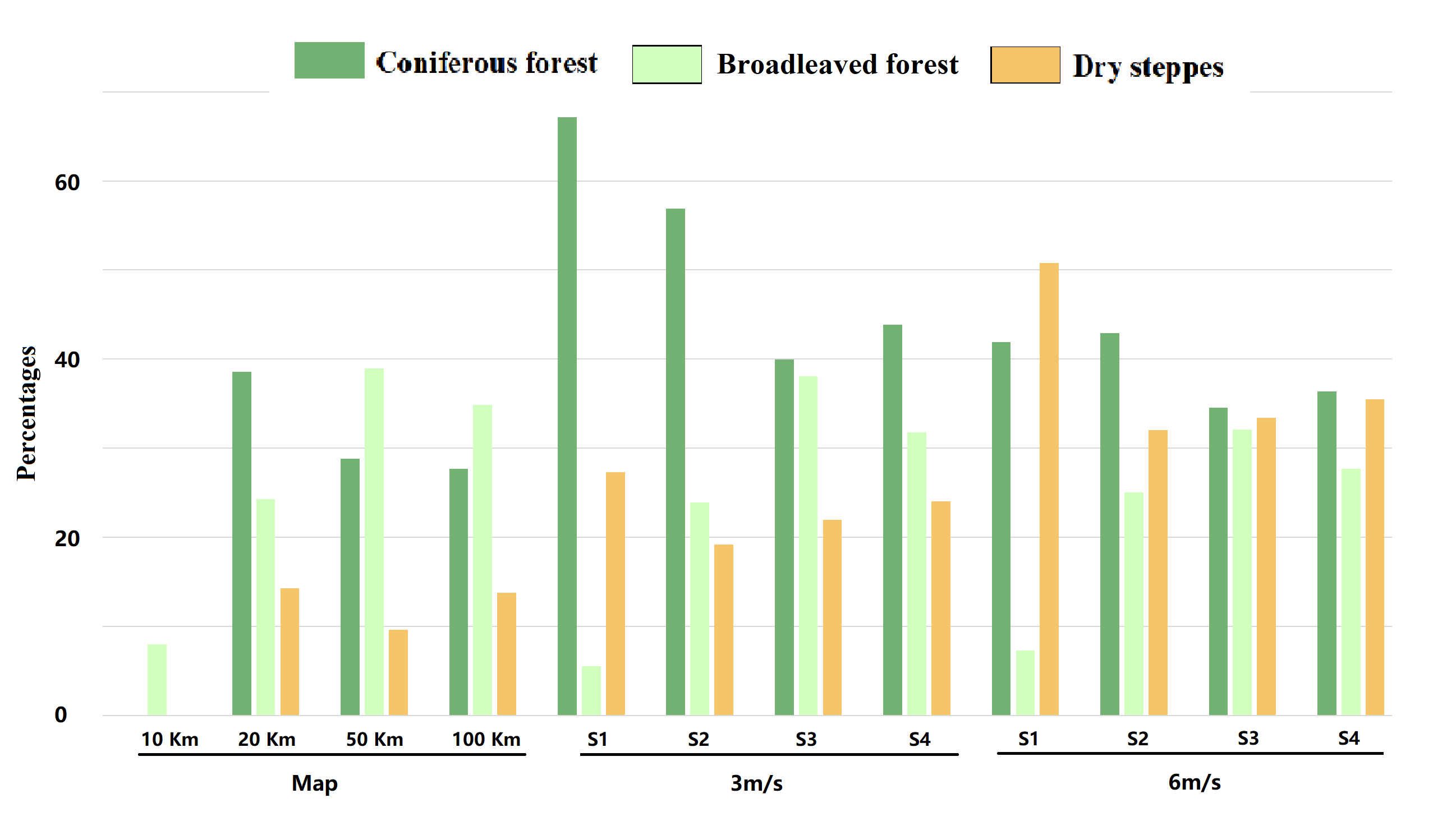


**Fig. S12. Marginal region:** Land cover of the three vegetation categories based on the 10, 20, 50, and 100 km maps versus REVEALS estimates. All REVEALS reconstructions correspond to a Zmax of 50 km. S1, S2, S3, and S4 are related to different RPP options used for the model runs. The two wind speed alternatives are also shown, i.e., 3 and 6 m/s. See the Methods section for more explanation about the model settings.
